# Dirac Surface‐State Driven Broad Spectral Band Low Quantum Energy Photoresponse in Quaternary Topological BiSbSe_2_Te

**DOI:** 10.1002/advs.202522592

**Published:** 2026-03-12

**Authors:** Tianning Zhang, Jinchao Tong, Jiantian Zhang, Peng Yu, Heng Luo, Luo Yan, Boyu Yang, Tiantian Huang, Junhui Ran, Yan Chen, Liujiang Zhou, Xin Chen, Ning Dai, Jianlu Wang, Junhao Chu

**Affiliations:** ^1^ School of Physics and Optoelectronic Engineering Hangzhou Institute for Advanced Study University of Chinese Academy of Sciences Hangzhou China; ^2^ State Key Laboratory of Infrared Physics Shanghai Institute of Technical Physics Chinese Academy of Sciences Shanghai China; ^3^ University of Chinese Academy of Sciences Beijing China; ^4^ Shanghai Frontiers Science Research Base of Intelligent Optoelectronic and Perception Institute of Optoelectronics Fudan University Shanghai China; ^5^ School of Materials Science and Engineering Sun Yat‐sen University Guangzhou China; ^6^ School of Electronic Information Central South University Changsha China; ^7^ School of Physics University of Electronic Science and Technology of China Chengdu China; ^8^ College of Materials Science and Engineering Hunan University Changsha China

**Keywords:** low quantum energy photons, photodetection, quaternary BiSbSe2Te, THz

## Abstract

2D topological materials are garnering significant interest for their potential in optoelectronic applications, particularly due to their unique quantum transport properties and exceptional optical characteristics. In this work, quaternary topological BiSbSe_2_Te with stoichiometry modulation from Bi_2_Se_3_ are synthesized and demonstrates a broad spectral band photoresponse, ranging from infrared to terahertz and to millimeter waves, with a particular excellence on detection of low quantum energy photons. The observed photoresponse is attributed to the excitation of plasmonic nonequilibrium electrons originating from the topological Dirac surface states inherent in the BiSbSe_2_Te. The detector exhibits remarkable performance, achieving a responsivity of 8174 V/W and a noise equivalent power of 4.7 × 10^−^^1^ ^3^ W/Hz^1/2^ at 0.094 THz, with a wide low quantum energy terahertz and millimeter wave photoresponse spanning from 0.032 to 0.173 THz. The study underscores the potential of Dirac surface‐state driven photoresponse in BiSbSe_2_Te, paving the way for the development of sensitive, broad spectral band and room‐temperature low quantum energy photodetectors, which are highly pursued for a variety of applications.

## Graphical abstract

Quaternary topological BiSbSe_2_Te are synthesized and demonstrates a broad spectral band photoresponse, ranging from infrared to terahertz and to millimeter waves, with a particular excellence on detection of low quantum energy terahertz photons. The observed photoresponse is attributed to the excitation of plasmonic nonequilibrium electrons originating from the topological Dirac surface states.

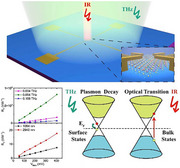

## 1 Introduction

Two‐dimensional (2D) materials, including graphene and a diverse array of van der Waals structures, have shown great promise in optoelectronic applications due to their unique properties, particularly in low quantum energy terahertz (THz) and millimeter wave (MmW) range [1, 2]. These materials are key to overcome the challenges in detecting THz/MmW signals, which have historically been constrained by a lack of efficient materials suitable for operation within the electromagnetic band [3, 4, 5, 6, 7]. Numerous THz/MmW detectors have been developed based on various effects such as bolometric [8], photothermoelectric [9], and plasma wave rectification [5] offering new possibilities for excellent low quantum energy photodetection.

Recent advancements in theoretical understanding and synthesis techniques have led to the discovery and fabrication of a variety of 2D topological materials, including topological insulators and semimetals [10, 11]. These materials, characterized by a gapless energy band structure with unique quantum transport behavior, are particularly promising for low quantum energy photodetection, distinguishing them from conventional optoelectrical 2D semiconductors that are limited by characteristic bandgaps. Consequently, a range of THz/MmW low quantum energy photodetectors have been realized using these materials [12, 13, 14, 15], alongside the observation of novel optoelectronic phenomena such as the photogalvanic effect [16], bulk photovoltaic effect [17] and nonlinear Hall effect [18].

Plasmons, which are collective oscillations of electrons coupled with photons, have gained significant attention in topological insulators. These insulators, with their insulating bulk and time‐reversal symmetry‐protected metallic surface, host surface plasmon polaritons (SPPs) formed by massless Dirac carriers [19, 20, 21]. Second‐generation topological insulators, such as Bi_2_Te_3_, Bi_2_Se_3_, and Sb_2_Te_3_, possess a single Dirac cone in their surface states [22], facilitating electron movement along the surface while restricting it through the bulk. The coupling of light with these topologically protected surface carriers can excite SPPs, leading to minimal scattering and enabling the exploration of novel photoresponses [23, 24].

In particular, Bi_2_Se_3_ has been studied extensively, with its Dirac plasmons demonstrating THz/MmW range excitations [20, 25]. These SPPs can decay either radiatively, through re‐emitted photons, or non‐radiatively, by transferring energy to nonequilibrium electrons via intraband or interband absorption [26, 27, 28]. This non‐radiative decay mechanism contributes to photocurrent or photovoltage responses within appropriately designed detector architectures [29, 30], thus overcoming the traditional limit where the quantum energy of a photon must exceed a characteristic transition energy to generate significant photoconductivity. This opens new avenues for developing sensitive, broad spectral band low quantum energy THz/MmW photodetectors that can operate at room temperature, addressing the current obstacles of low sensitivity, narrow spectral bandwidth, and cryogenic operation requirements.

The excitation of 2D Dirac surface plasmons in topological materials is sensitive to carrier density oscillations [31, 32, 33], influencing material selection. Topological insulator such as Bi_2_Se_3_ can support SPPs that are formed by massless Dirac carriers [20, 25, 34]. Quaternary (Bi,Sb)_2_(Se,Te)_3_ trichalcogenide compounds are reported to support SPPs across a broad spectral range due to inter‐ and intra‐surface band transitions [35]. Moreover, it has been reported that SPPs can be realized not only at the interface between a topological insulator and a metal but also at the interface between vacuum and a doped topological insulator with a nonvanishing bulk carrier density [36]. The modulation of stoichiometry from simple binary compounds can further alter the excitation of surface‐state‐related plasmons [37, 38], presenting opportunities to enhance photodetection capabilities and trigger new optoelectrical behaviors.

In this work, a layered quaternary topological material, BiSbSe_2_Te, has been synthesized with precisely controlled composition ratios, given that it has the smallest effective mass of electrons among all quaternary (Bi,Sb)_2_(Se,Te)_3_ trichalcogenide compounds [35]. BiSbSe_2_Te photodetectors capable of responding to both infrared (IR) and low quantum energy THz/MmW photons have been designed and characterized over a broad spectral band ranging from 1064 to 9.4 mm. In the IR range, the detector achieves moderate responsivities of 7.6 V/W at 1064 nm and 17.8 V/W at 2940 nm. In the low quantum energy photon range, the BiSbSe_2_Te detector demonstrates broad spectral band sensitivity and room‐temperature operation capability from 0.032 to 0.173 THz, spanning from K_a_ to W and to D bands. Notably, it exhibits a responsivity (R_V_) of 8174 V/W, a noise equivalent power (NEP) of 4.7 × 10^−13 ^W/Hz^1/2^, a detectivity (D^*^) of 1.8 × 10^11^ cmHz^1/2^/W, and a response speed of 21.7 µs at 0.094 THz. This exceptional performance is attributed to the excitation of plasmonic nonequilibrium electrons within the topological surface states of BiSbSe_2_Te, positioning it competitively among state‐of‐the‐art technologies for broad spectral band low quantum energy photodetection.

## 2 Results and Discussion

### 2.1 Crystal Growth and Characterization

**FIGURE 1:**
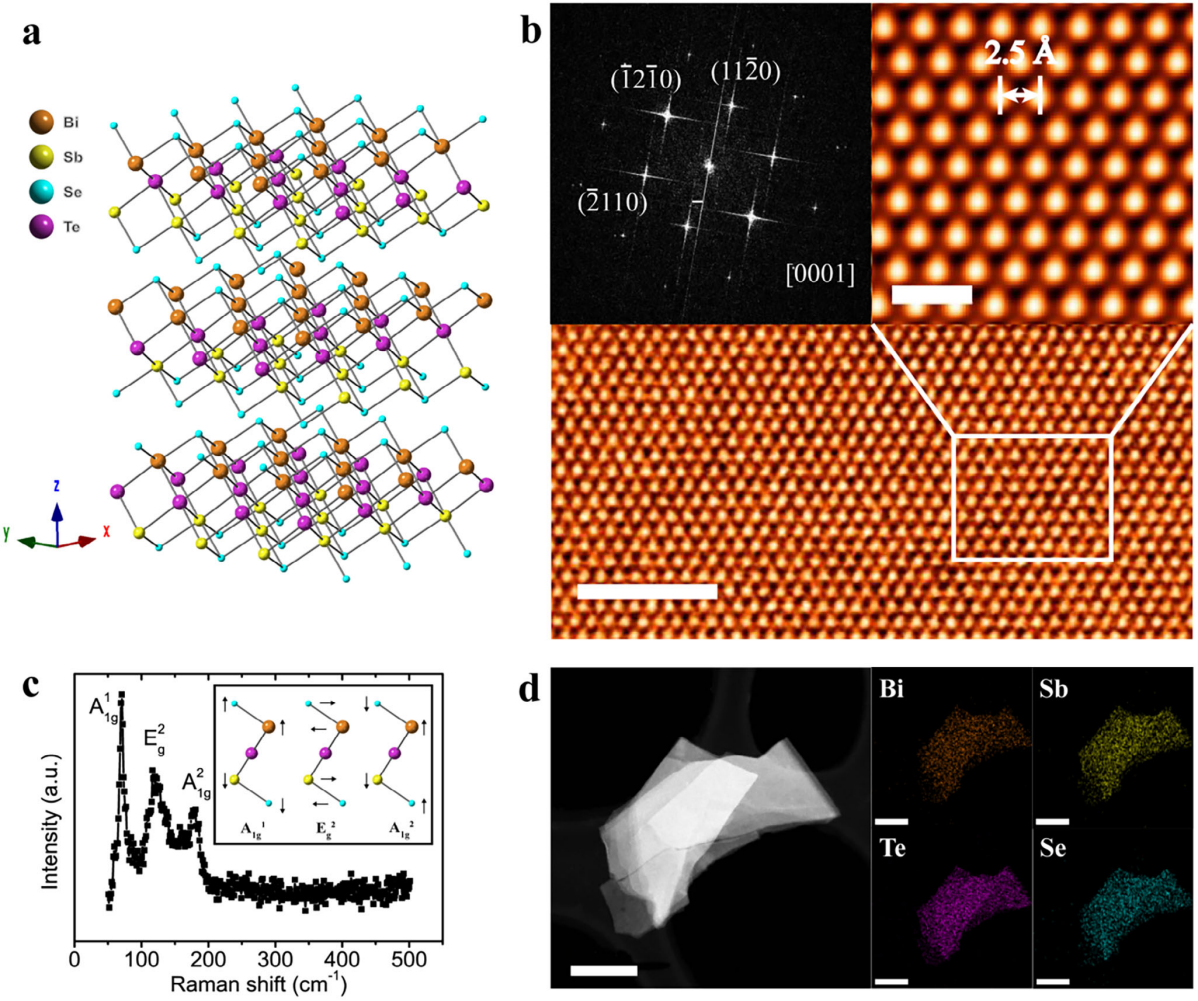
Structure and characterization of BiSbSe_2_Te. (a) Crystal structure of BiSbSe_2_Te. Brown, green, blue and purple balls denote Bi, Sb, Se and Te atoms, respectively. (b) Scanning transmission electron microscopy (STEM) image of a typical BiSbSe_2_Te flake. The scale bar is 2 nm. The scale bar of the zoom‐in image is 0.5 nm. The inset image is the electron diffraction patterns taken along the direction of [0001]. (c) Raman spectrum of BiSbSe_2_Te flakes, measured using a 532 nm laser, shows multiple peaks at 70, 120, and 180 cm^−1^, corresponding to A_1g_^1^, E_g_^2^, and A_1g_^2^ vibrational modes, respectively. The inset figure shows the vibrational modes. (d) Scanning electron microscope (SEM) image of a typical BiSbSe_2_Te flake and its corresponding elemental mapping analysis of Bi, Sb, Te and Se elements. The scale bar is 500 nm.

Bulk BiSbSe_2_Te crystallizes in the rhombohedral space group R $\bar{3}$ m (Figure 1a) and constitutes stacks of quintuple layers (QLs) by covalent bonds in the sequence of Se1─Bi─Te─Sb─Se2, which are further bonded together with weak van der Waals interactions. In our experiment, single‐crystalline ingots of BiSbSe_2_Te were synthesized by solid‐state reaction and were characterized by scan transmission electron microscope (STEM) (see Methods). A clear hexagonal lattice in Figure 1b and a six‐fold symmetry [0001] zone axis pattern in the inset of Figure 1b cooperatively confirms the high quality of BiSbSe_2_Te. The measured crystalline atom spacing d equals to 2.5 Å, which indicates that the cell parameters a = 4.3 Å. The crystal structure of BiSbSe_2_Te was further verified by Raman spectroscopy. The Raman spectrum (Figure 1c) shows three characteristic peaks at 70, 120 and 180 cm^−1^, corresponding to $A_{1g}^{1}$, $E_{g}^{2}$ and $A_{1g}^{2}\;$ modes, respectively (Figure 1c, inset). The X‐ray powder diffraction (XRD) pattern further confirms the structure of BiSbSe_2_Te (Figure S1a), where all diffraction peaks can be indexed based on the rhombohedral structure with R $\bar{3}$ m space group. BiSbSe_2_Te shares the same structure as its members Bi_2_Te_3_, Bi_2_Se_3_, with cell parameters a = 4.3 Å, c = 30.4 Å, which corresponds to the distance of interplanar planes. To confirm the chemical composition, energy dispersive X‐ray spectrum (EDS) and elemental mapping images (Figure 1d) were obtained. The peaks of Bi─M, Sb─L, Se─L and Te─L can be identified clearly in EDS (Figure S1b), while elemental mapping of these elements reflects a uniform distribution. The EDS analysis shows an atomic ratio of Bi_1.09_Sb_0.91_Se_2_Te (Figure S1c,d), which is very close to the growth design of 1:1:2:1. To further confirm the ratio, we also evaluated the crystal by X‐ray photoelectron spectroscopy (XPS). As shown in Figure S2, a result of Bi_1.2_Sb_1.01_Se_1.79_Te was derived, which is consistent with that by EDS. The broadband low quantum photon absorption was confirmed at 0.1–2 THz (Figure S3), which is similar with the Dirac plasmonic absorption in Bi_2_Se_3_ [39].

### 2.2 Detector Design, Fabrication and Characterization for THz/MmW and IR

To take an insight on the optoelectronic properties, BiSbSe_2_Te flake was exfoliated and transferred onto SiO_2_/Si substrate. Then, an antenna‐assisted detector was fabricated with BiSbSe_2_Te flake located in the gap (see Methods). The dipole‐like antenna was used to improve coupling of low quantum energy THz/MmW photons as their wavelength is much longer than the lateral dimension of BiSbSe_2_Te flake. Long wavelength/low quantum energy photons would be largely scattered if there is no such couple design. The rotationally symmetrical four terminal detector was fabricated on a p++ Si wafer with a 280 nm dielectric SiO_2_ layer. 10 nm Ti/100 nm Au electrodes were deposited by electron beam evaporation after standard lithography. Such four‐terminal configuration can help to achieve polarization‐independent detection of low quantum energy photons along *x*‐ and *y*‐ axis, enabling capability of multiple‐dimensional information acquisition.

**FIGURE 2:**
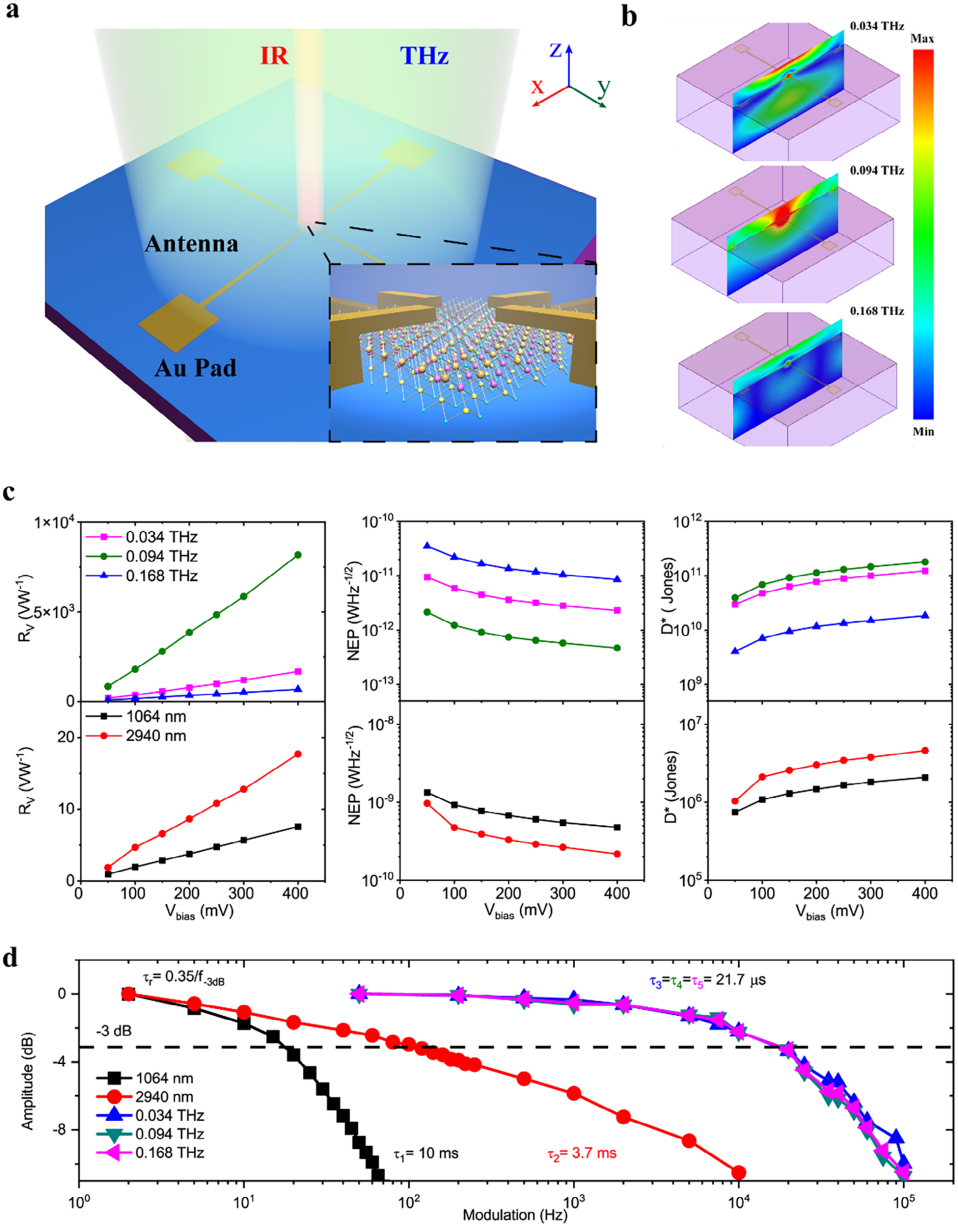
BiSbSe_2_Te detector design and performance. (a) Schematic diagram of the detector structure. The infrared radiation incidents directly onto the BiSbSe_2_Te flake, while the THz/MmW radiations are coupled into the flake by the antenna. (b) Distribution of simulated optical field for BiSbSe_2_Te detector at 0.034, 0.094, and 0.168 THz, respectively. (c) R_V_, NEP, and D^*^ as a function of the electrical bias at room temperature under the incident sources with different frequencies. The power of the incident light is 10.47 Wcm^−2^ at 1064 nm, 0.308 Wcm^−2^ at 2940 nm, 0.00151 Wm^−2^ at 0.034 Hz, 0.00151Wm^−2^ at 0.034 THz, 0.00143Wm^−2^ at 0.094 THz, 0.0328 Wm^−2^ at 0.168 THz, separately. (d) Amplitude–frequency response of the detector under the incident sources with different frequencies.

In detector design, except low quantum energy THz/MmW photons, we also check the optoelectrical performance of BiSbSe_2_Te to IR photons. Figure 2a schematically illustrates the detector with incident radiations for both THz/MmW and IR. The BiSbSe_2_Te flake serves as active material in the channel for both IR and THz/MmW photons. Four electrodes as well as antennas are designed to couple low quantum energy THz/MmW radiation [40]. The morphology of the antenna was precisely designed to achieve the best coupling of photons with specific energy within THz/MmW range. In our design, the antenna parameters (Figure S4) a, d1, d2, L equal to 150, 2.5, 20, and 759 µm, respectively. As such, the antenna serves as a half‐wave dipole‐like antenna for optimized coupling frequency of ∼0.094 THz. The integration of the antenna will not disturb the absorption of IR wave as for this band, the wavelength is quite smaller than the lateral dimension of the active BiSbSe_2_Te flake and the antenna just serves as extension of the metallic contacts. Based on the configuration above, broad spectral band absorption would occur as the active BiSbSe_2_Te directly absorbs infrared photons and indirectly for the long wavelength low quantum energy photons via the antenna. Optical and atomic force microscopy (AFM) images demonstrate good contact between the metal and the electrodes (Figure S4). The measured thickness of the BiSbSe_2_Te flake is 98 nm from AFM, which means there are 98 quintuple layers. The typical coupling of the antenna was simulated by High Frequency Structure Simulator (HFSS). Here, we present the typical distribution of the optical field under illumination of THz/MmW radiations at frequencies of 0.034, 0.094, and 0.168 THz, respectively (Figure 2b). As shown, the antenna can concentrate incident radiations around the antenna gap, where large optical field enhancement is observed. The antenna is capable of coupling low quantum energy THz/MmW photons into the active BiSbSe_2_Te flake. The simulation also shows that the designed antenna has best coupling efficiency at 0.094 THz, followed by 0.034 THz and 0.168 THz, indicating best detecting performance at 0.094 THz.

We first measured dark current–voltage (*I*–*V*) curves of the BiSbSe_2_Te detector for both polarization directions (Figure S5a). Good repeatability, linearity and symmetry demonstrate good ohmic contact and therefore excludes the potential rectification from barriers, which forms the detection for THz/MmW Schottky diode and other rectifying diodes. To evaluate the performance, several Figures of merit are used, such as photoresponsivity (R_V_), NEP, and D^*^ (See methods). Figure 2c shows the measured R_V_, NEP, and D^*^ of the detector at different voltage bias V_bias_ for room temperature operation at K_a_ band (0.034 THz, 8.8 mm), W band (0.094 THz, 3.2 mm), D band (0.168 THz, 1.8 mm), NIR band (1064 nm), and MWIR band (2940 nm). As presented, the detector demonstrates extremely broad spectral band detection from infrared to THz and to millimeter wave range. For all the bands, R_V_ increases linearly with voltage bias as the collected carriers by electrodes are proportional to the drift velocity, which is linearly proportional to external voltage bias within unsaturated range. This nature is the same as a typical photoconductor. NEP and D^*^ show corresponding improvement while increasing bias, but due to increment of dark current shot noise level *i*_n_ = (2qI_d_)^1/2^ (see Methods and Figure S6a), they tend to saturate at a relatively large bias of 400 mV. It is also observed that the detector has enormous differences in performance for infrared and low quantum energy THz/MmW range. For infrared wave, responsivities are 7.6 and 17.8 VW^−1^ with a bias of 400 mV for the wavelength of 1064 nm (NIR) and 2940 nm (MIR), respectively. And the corresponding NEP are 4.8 × 10^−10^ WHz^−1/2^ and 2.2 × 10^−10^ WHz^−1/2^. Under the same bias, Detectivity (D^*^) of 2.1 × 10^6^ cmHz^1/2^W^−1^ and 4.6 × 10^6^ cmHz^1/2^W^−1^ are achieved, respectively. Such moderate performance for infrared wave is not as good as other 2D material based infrared detectors, which have been summarized in recent reviews [41, 42]. But at the low quantum energy photon range, a responsivity of 8174 VW^−1^ is achieved for 0.094 THz when the detector operates under the same bias of 400 mV. The corresponding NEP and detectivity (D^*^) are 4.7 × 10^−13^ WHz^−1/2^ and 1.8 × 10^11^ cmHz^1/2^W^−1^, respectively. The R_V_, NEP, D^*^ can still reach to 1680 VW^−1^, 2.3 × 10^−12^ WHz^−1/2^, 1.2 × 10^11^ cmHz^1/2^W^−1^ and 685 VW^−1^, 8.5 × 10^−12^ WHz^−1/2^, 1.9 × 10^10^ cmHz^1/2^W^−1^ at 0.034 THz and 0.168 THz, respectively, although the antenna is not optimized for these two frequencies. Such performance is comparable to state‐of‐the‐art technologies (Table S1). The response speed or rise time (signal from 0 to 63.2 of the maximum) of the detector can be derived from *t*_r_ = 0.35/*f*_‐3 dB_ (*f*_‐3 dB_ is the −3 dB bandwidth). To characterize it, we measured the amplitude‐frequency response (Figure 2d) and verified it by oscilloscope at THz range (Figure S6b). As shown, the detector exhibits significant difference for radiation between IR and THz/MmW. At low quantum energy range, it is ∼21.7 µs for 0.034 THz, 0.094 and 0.168 THz, which is much faster than that for infrared wave range (10 ms for 1064 nm and 3.7 ms for 2940 nm).

### 2.3 Surface‐State Driven Photoresponse

To gain a deep insight into the excellent photoresponse of BiSbSe_2_Te detector for low quantum energy photons, as well as the difference with IR bands, we first focus on the structure design of the detector. As shown in Figure 2a, the dipole‐like antenna is symmetric, which ensures symmetric coupling of THz/MmW photons onto the active BiSbSe_2_Te flake, resulting in same heating of two metal‐BiSbSe_2_Te contacts and symmetric heating within BiSbSe_2_Te flake. This excludes the contribution from photothermoelectric effect arising from temperature gradient or difference in Seebeck coefficient [9, 43, 44]. Besides, as we observed symmetric and linear *I*–*V* character, the contribution of rectify effect can be neglected [6, 45]. Meanwhile, as electrodes and photocurrent in the same horizontal plane, Dember effect generated from different diffusion speed of carriers in the vertical direction can also be excluded [45, 46]. Another effect would generate photoresponse is the bolometric effect [47] in BiSbSe_2_Te. If we assume all the incident low quantum energy photons are absorbed by the material, the estimated bolometric response at THz range is only about 0.077 VW^−1^ (See Method), which is 4–5 orders lower than our measured response (8174 VW^−1^) in Figure 2. Therefore, bolometric contribution to the response can be neglected at THz range. However, at infrared wavelength, depending on the differences in their response times, the responsivity will increase by 2 to 3 orders of magnitude (7.7–77 V/W), which is comparable with the value we measured (17.8VW^−1^). It indicates that due to the excessively narrow bandgap of the material, the main contribution to the infrared response is comparable with the calculated bolometric effect.

**FIGURE 3:**
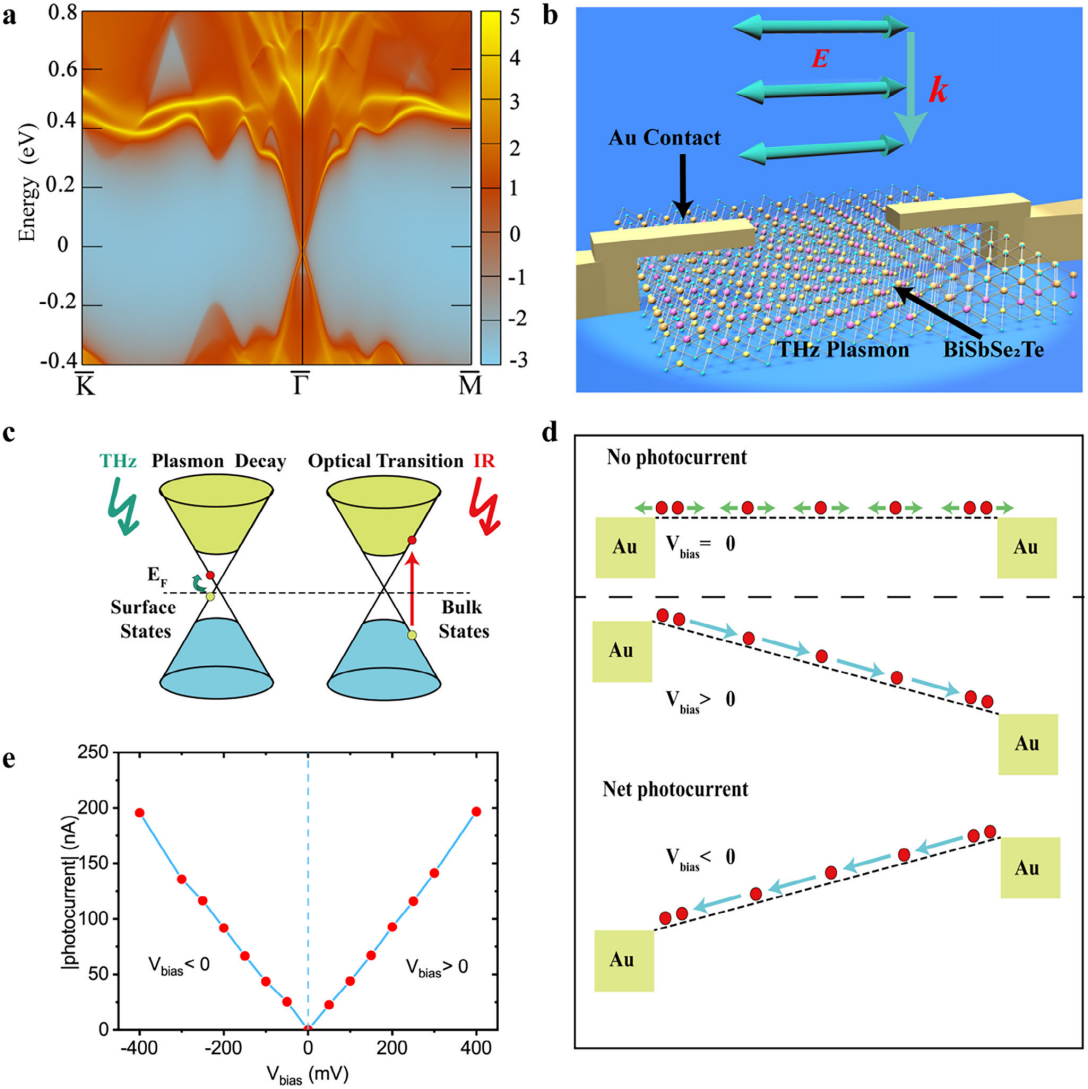
Detection mechanism analysis. (a) Calculated surface band structure of BiSbSe_2_Te. (b) Schematic of the BiSbSe_2_Te photodetector under THz radiation. Blue arrows denote the TM polarization orientation of incident THz wave. The light blue round shadow denotes the generation of Dirac SPPs around surface states. (c) Schematic illustration of the carrier transition under the THz/infrared source. The red balls denote electrons, and the yellow balls denote holes. (d) The movement of the nonequilibrium carriers without/with electrical bias under excitation of Dirac SPPs. (e) Photocurrent response of BiSbSe_2_Te with different voltage direction.

We attribute the excellent low quantum energy photoresponse to the unique properties of BiSbSe_2_Te. To investigate the electronic band structure and optical properties of the material, we performed first‐principle calculation based on the lattice parameters we obtained, with and without accounting for spin–orbital coupling (Figure S7). Our calculation points out that BiSbSe_2_Te exhibits topological insulator behavior, in which the strong spin–orbit coupling leads to a band inversion and the appearance of topologically protected surface states in the bandgap (Figure 3a; Figure S7). As shown in Figure 3a, the calculated surface band structure of BiSbSe_2_Te reveals Dirac‐like linear dispersions near the Fermi level, suggesting the presence of topologically protected surface states which may interact with low‐energy THz photons through enhanced surface absorption. This is in consistent with other reports [48, 49]. The broadband responsivity observed in the low‐frequency regime (0.1–2 THz) may be partly attributed to the collective excitation of surface carriers, potentially resembling Dirac plasmon‐like behavior, as seen in similar topological insulators.(Figure 3b) [3, 5, 6, 7, 20, 45]. However, without direct confirmation from spectroscopic or near‐field techniques, we acknowledge that Drude‐type absorption by free carriers in surface or bulk states cannot be ruled out. It is worth noting that the temperature‐dependent resistance of the BiSbSe_2_T shows a metallic linear trend $R=R_{\begin{array}{c} rt \end{array}}\;\left[1+\alpha (T-T_{0})\right]$, where α is the positive thermal coefficient (α = 0.004K^−1^), which suggests that the metallic surface states start to provide a dominant contribution to the overall conductivity [50]. Since the optical phonon energies of both the analogue compound BiSbSeTe_2_ flake (5.0–24.8 meV) [51] and SiO_2_ substrate (55.8–148.8 meV) [52] locate far away from the interest low quantum energy range (0.12–0.58 meV), plasmonic decay through emission of optical phonon can be neglected. The plasmons will decay through intraband absorption within the surface states owing to the low quantum energy character of THz/MmW photons. This helps to build up an excess carrier population in the Dirac cone via interband scattering processes involving bulk valence and conduction states [53], leading to generation of nonequilibrium electrons that are not in thermal equilibrium with atoms [3, 5, 6, 7, 45] (Figure 3c). Under zero bias (Figure 3d), the movement of these nonequilibrium electrons on the surface will mainly follow the law of random thermal motion (or omnidirectional scattering) and temperature‐dependent thermal diffusion, thus, there would be no net photocurrent formed within the detector. However, under external bias, the flat energy band will be tilted. Although the nonequilibrium electrons will keep their thermal motion, they will at the same time drift to the side with low potential under the electrical field force. The direction of the drift depends on that of the bias, resulting in direction consistency between photocurrent and bias voltage. Such unidirectional flow of nonequilibrium electrons will form net photocurrent in the detector. The photocurrent *I*_ph_ arising from surface states can be expressed as Iph  =  An(x)qµξ, where *A* is cross‐sectional area of suface layer, *n*(*x*) is the nonequilibrium electron density, *q* is elementary charge, *µ* is electron mobility and *ξ* is the electrical field which increases linearly with external bias. Therefore, linearly and symmetrically increased photocurrent response was observed for both positive and negative biases (Figure 3e).

The energy band structure of BiSbSe_2_Te and other topological insulators holds a distinctive linear energy‐momentum relationship, which leads to small effective mass since carriers behave as Dirac fermions [54, 55]. These nontrivial topological states of BiSbSe_2_Te associated with an exotic quantum transport have been demonstrated to show ultrafast charge transport after suddenly driving the system out‐of‐equilibrium [56], providing the flake metallic conduction at the surface. As a result, nonequilibrium carriers induced by surface states show higher carrier mobility, leading to better performance. For the infrared range, the photons are absorbed directly through bulk states and since the photon energy (1.17 eV@1064 nm, 0.42 eV@2940 nm) is larger than the bandgap (0.16 eV, see Figure 3c; Figure S7b), optical transition of bulk states would dominate. In such cases, the detector suffers from longer decay time as well as lower collection efficiency of the excited bulk states. Therefore, the photoresponse in the IR range is not as good as that in low quantum energy THz/MmW range. The higher carrier mobility (charge transport) of surface states for low quantum energy wave also results in faster response speed. In fact, the response time of these Dirac semimetal THz photo detectors maybe even shorter, due to their gapless band structures and high‐mobility Dirac fermions [10, 11], The trapping and recombination dynamics together with Bulk transport may make the photoresponse slower. Thickness optimization and heterostructure design may improve response speed in future studies.

**FIGURE 4:**
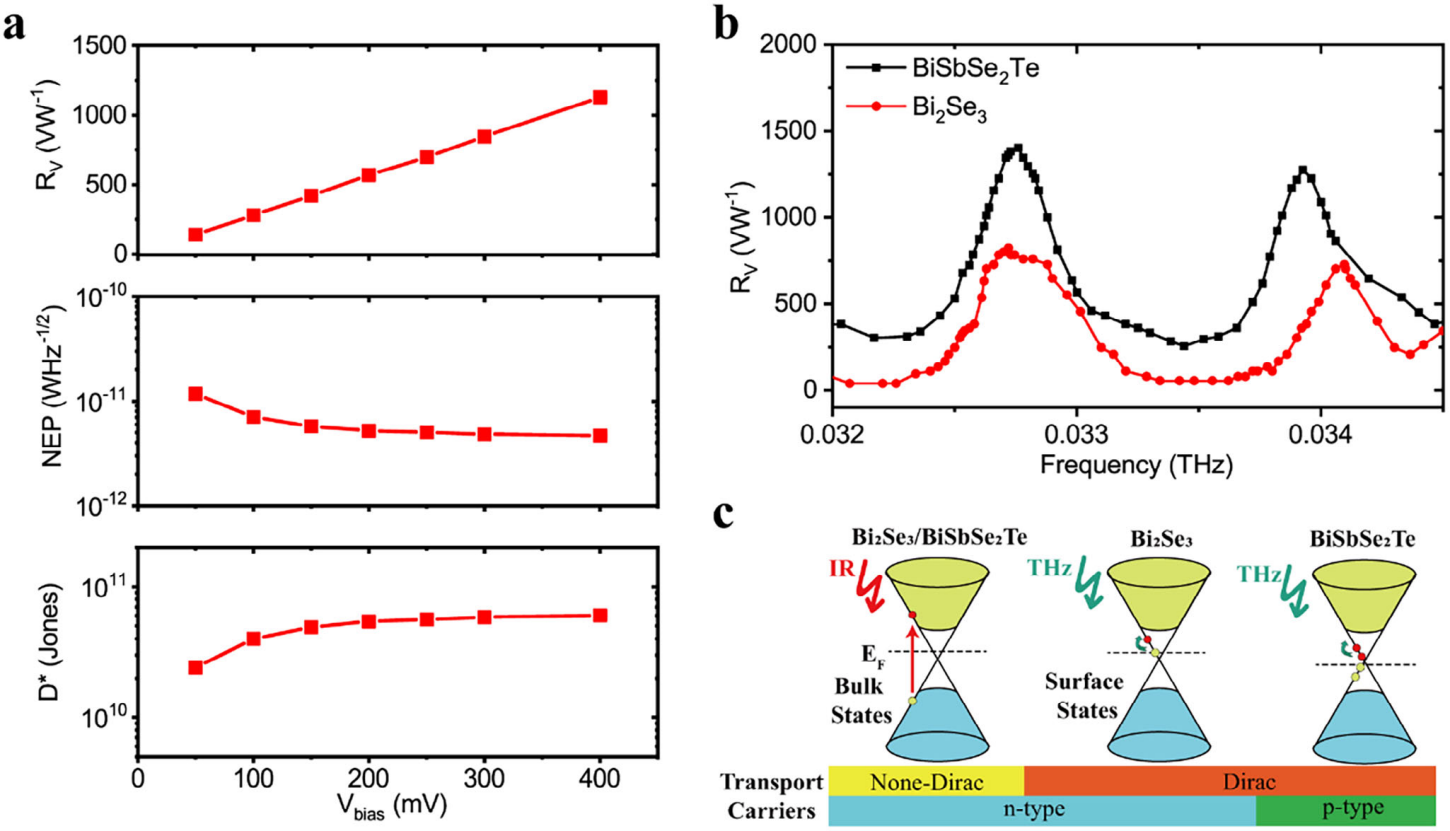
Comparison between BiSbSe_2_Te and Bi_2_Se_3_. (a) R_V_, NEP, and D^*^ of the Bi_2_Se_3_ detector at 0.034THz with different voltage bias. (b) Photocurrent response of BiSbSe_2_Te and Bi_2_Se_3_ in a frequency range of 0.032–0.0345 THz under a voltage bias of 400 mV. (c) Schematic illustration of the transport carrier properties and band diagram under the THz/infrared source. The red balls denote electrons, and the yellow balls denote holes.

To understand more about the contribution of surface state to low quantum energy photoresponse, we also fabricated binary topological Bi_2_Se_3_ detector with the same configuration and observed similar photocurrent response to low quantum energy photons (Figure 4a,b), but the performance is not as good as that of BiSbSe_2_Te (Figure S8). For example, under 400 mV bias, Bi_2_Se_3_ detector only demonstrates R_v_, NEP, D^*^ of 1120 V/W, 4.6 × 10^−12^ WHz^−1/2^, 5.8 × 10^10^ cmHz^1/2^W^−1^, which lags behind its quaternary BiSbSe2Te counterpart. This is because BiSbSe_2_Te has larger density of surface state at the vicinity of Dirac point near Fermi level compared to Bi_2_Se_3_ (Figure S7c,f), resulting in more nonequilibrium electrons under excitation of low quantum energy photons. Band structure calculations show that the chemical potential in Bi_2_Se_3_ is always located in the bulk bandgap, whereas the Dirac cone dispersion changes systematically so that the Dirac point moves up in energy with doping. The content of Sb leads to a sign change of Dirac carriers from n‐ to p‐type (Figure 4c), which are confirmed by both our calculation results and angle‐resolved photoemission spectroscopy [49]. Bi_2_Se_3_ is confirmed to be a semiconductor with a bandgap of 0.26 eV, while BiSbSe_2_Te has narrower bandgap (0.16 eV) and behaves like a highly degenerate semiconductor [50] (Figure S7), introducing a higher density of state near the Fermi level. The stoichiometry modulation from binary Bi_2_Se_3_ to quaternary BiSbSeTe_2_ offers more possibilities for coupling between surface states and low quantum energy photons [57]. Thus, more nonequilibrium carriers will be induced during the decay process [26, 27, 28, 29, 30]. Moreover, smaller effective mass in quaternary topological insulator has been demonstrated [48], which may lead to higher carrier mobility, thus faster drift velocity and more collected nonequilibrium carriers.

### 2.4 Broad Spectral Band Low Quantum Energy THz/MmW Photoresponse

**FIGURE 5:**
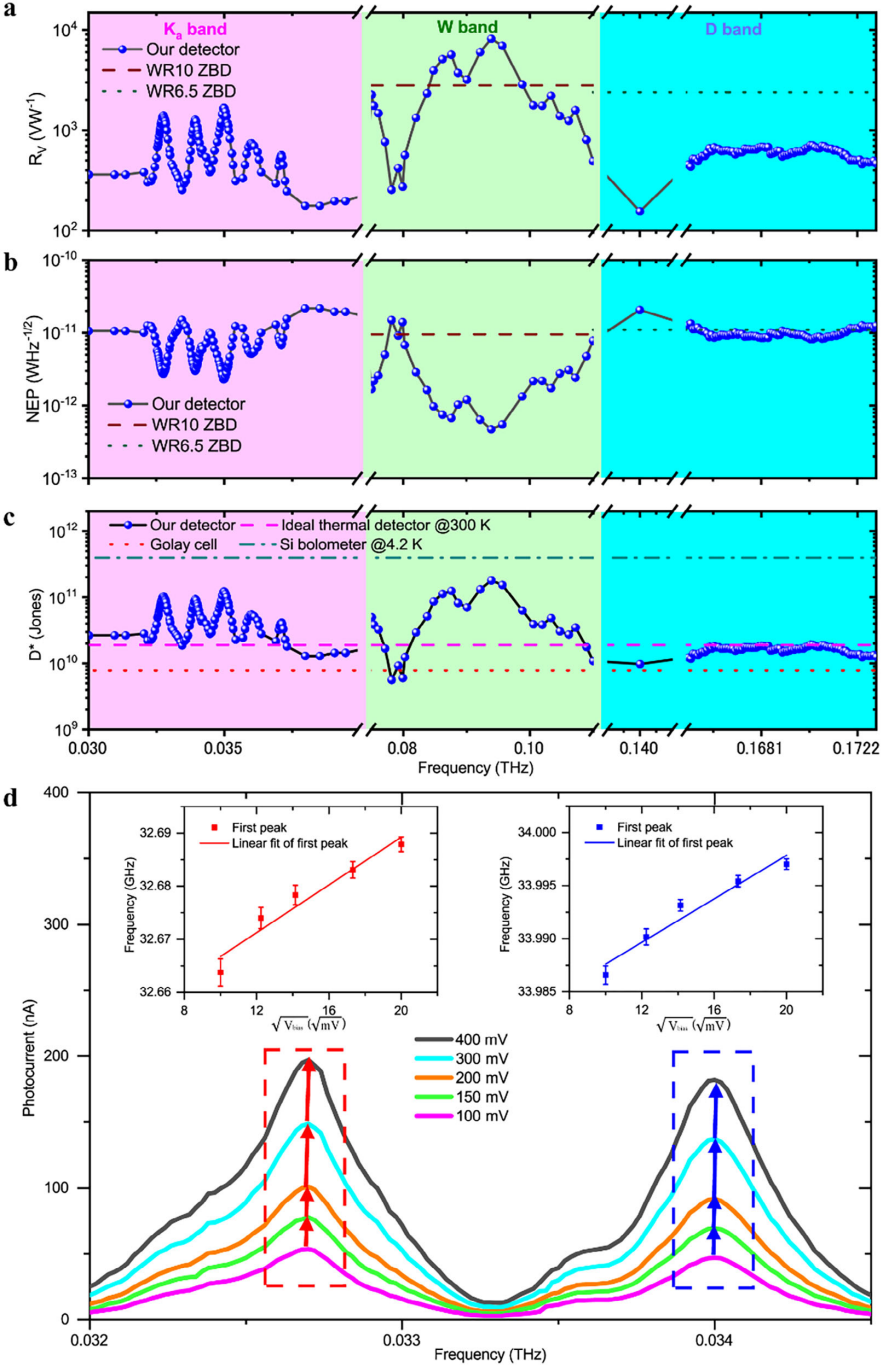
Spectral response in THz/MmW range. (a–c) R_V_, NEP, and D^*^ of the detector in a frequency range of 0.032–0.173 THz under a voltage bias of 400 mV. The dash lines in (a) and (b) represent typical performance of state‐of‐the‐art commercial zero‐bias SBDs (WR10 ZBD and WR6.5 ZBD) from VDI with the frequency range from 0.075 to 0.173 THz. The dash lines in c represent D^*^ of an ideal thermal‐type detector at room temperature, Golay cell and an Si bolometer at 4.2 K. (d) The peak positions modulation under different bias. The insert figures show the relationship between peak position and bias voltage.

Owing to the good photoresponse of BiSbSeTe_2_ detector to low quantum energy photons, we then characterized the spectral response of the detector within a broad range from K_a_ (0.0265–0.04 THz) to W (0.075–0.11 THz) and to D (0.11–0.17 THz) band. Each of these bands is critical for supporting high‐frequency, high‐bandwidth and large capacity communication, especially in fields like satellite communications, 5G/6G networks, radar systems, and scientific exploration. As shown in Figure 5a–c, the detector shows its best performance for W band with peak R_V_, NEP, and D^*^ of 8174 VW^−1^, 4.7 × 10^−13^ WHz^−1/2^, and 1.8 × 10^11^ cmHz^1/2^W^−1^, respectively at 0.094 THz, agreeing well with the single‐frequency test in Figure 2. Such performance is superior to the best commercially available zero‐bias SBDs (WR10 ZBD, R_V_ = 2800 VW^−1^, NEP = 9.5 × 10^−12^ WHz^−1/2^) from VDI [58]. For the D band, our detector also demonstrates very close NEP (8.5 × 10^−12^ WHz^−1/2@0.170^ THz) with WR6.5 ZBD (1.1 × 10^−11^ WHz^−1/2^) although the responsivity is lower. For K_a_ band, currently, there is no available counterpart from VDI. Our detector demonstrates R_V_, NEP, and D^*^of 1680 VW^−1^, 2.3 × 10^−12^ WHz^−1/2^, and 1.2 × 10^11^ cmHz^1/2^W^−1^ at 0.034 THz. In Figure 5c, D^*^ of an ideal thermal detector [45], a Golay cell (room temperature operation), and a Si bolometer (4.2 K operation) are plotted. Our detectors demonstrate a general 1–2 orders of magnitude improvement in performance in a broadband range than the ideal thermal detector and the Golay cell. At the best point of 0.094 THz, the performance is even comparable to the cooled Si bolometer. Importantly, the broad spectral band photoresponse observed above is realized by a single detector, which, however, requires multiple modules for ZBD technology. For our strategy, by optimizing the design of the couple antenna, we can obtain even better performance at specific frequencies. As an example, a bowtie antenna was designed and optimized for a BiSbSeTe_2_ detector for 0.168 THz radiation (Figure S9). Under the same voltage bias of 400 mV, the detector demonstrates R_v_, NEP and D^*^ of 1039 V/W, 3.09 × 10^−12^ WHz^−1/2^, and 5.54 × 10^10^ cmHz^1/2^W^−1^, respectively, which are better than those of the dipole‐like antenna detector for the same frequency as shown in Figure 5 (R_v_ = 685 V/W, NEP = 8.50 × 10^−12^ WHz^−1/2^ and D^*^ = 1.85 × 10^10^ cmHz^1/2^W^−1^). In experiments, we observed multiple resonance peaks, for example, those peaks at 0.088, 0.035, 0.033, and 0.034 THz. Such character in the spectral response is due to multiple internal reflection from the substrate interface (or excitation of substrate modes), which can vary the antenna pattern (gain and input impedance) [59]. This character might be useful for future application of frequency comb spectroscopy [60].

Notably, (Bi,Sb)_2_(Se,Te)_3_ topological material exhibits an intrinsic plasmonic resonance at ∼2 THz [19, 20, 21]. Direct observation of plasmonic signatures at this frequency in our photodetection measurements was precluded by excitation source limitations, as our system only operates at 20–40 GHz and ∼170 GHz, lacking a tunable high‐power terahertz source for the 1–2 THz range. Terahertz time‐domain spectroscopy (TDS) measurements (see Figure S3) verified a strong absorption band at 1–1.5 THz for the material, a spectral signature of its intrinsic plasmonic behavior. Our device's antenna coupling structure enables the effective exploitation of the material's intrinsic plasmonic effect even at the measured low frequencies, despite the plasmonic resonance being centered at the higher 2 THz band.

We further studied the spectral response under different bias. A very interesting blueshift of the response peak while increasing the bias was observed. As an example, the variation rule of peak response in K_a_ band is shown in Figure 5d. It has been demonstrated that terahertz plasmons can be tuned by temperature change [61]. Consequently, Joule heating which will induce temperature change should also be able to modulate the plasmonic response. When a current passes through the detector, the plasmon frequency will have a shift, as demonstrated in the insert of Figure 5d. By linear fitting, the shift of peak frequency is in proportion to the square root of bias voltage (V_bias_). In the low frequency regime, the Dirac plasmon frequency scales as $\sqrt[{4}]{D}$, where D is the Drude weight and D∝T (T is the temperature). And in Joule heating, T∝V_bias_^2^. Thus, in the end, the peak frequency shows a linear relationship with $\sqrt{V_{bias}}$. This phenomenon is in consistant with the blueshift of the response spectrum observed in graphite [61]. The derived shift rate is 22.5 GHz/ $\sqrt{V}$ and 10.2 GHz/ $\sqrt{V}$ for the peak near 32.66 and 33.98 GHz respectively. This bias tunable shifting would be very promising in developing very fine low quantum energy spectral combs [62].

## 3 Conclusion

**FIGURE 6:**
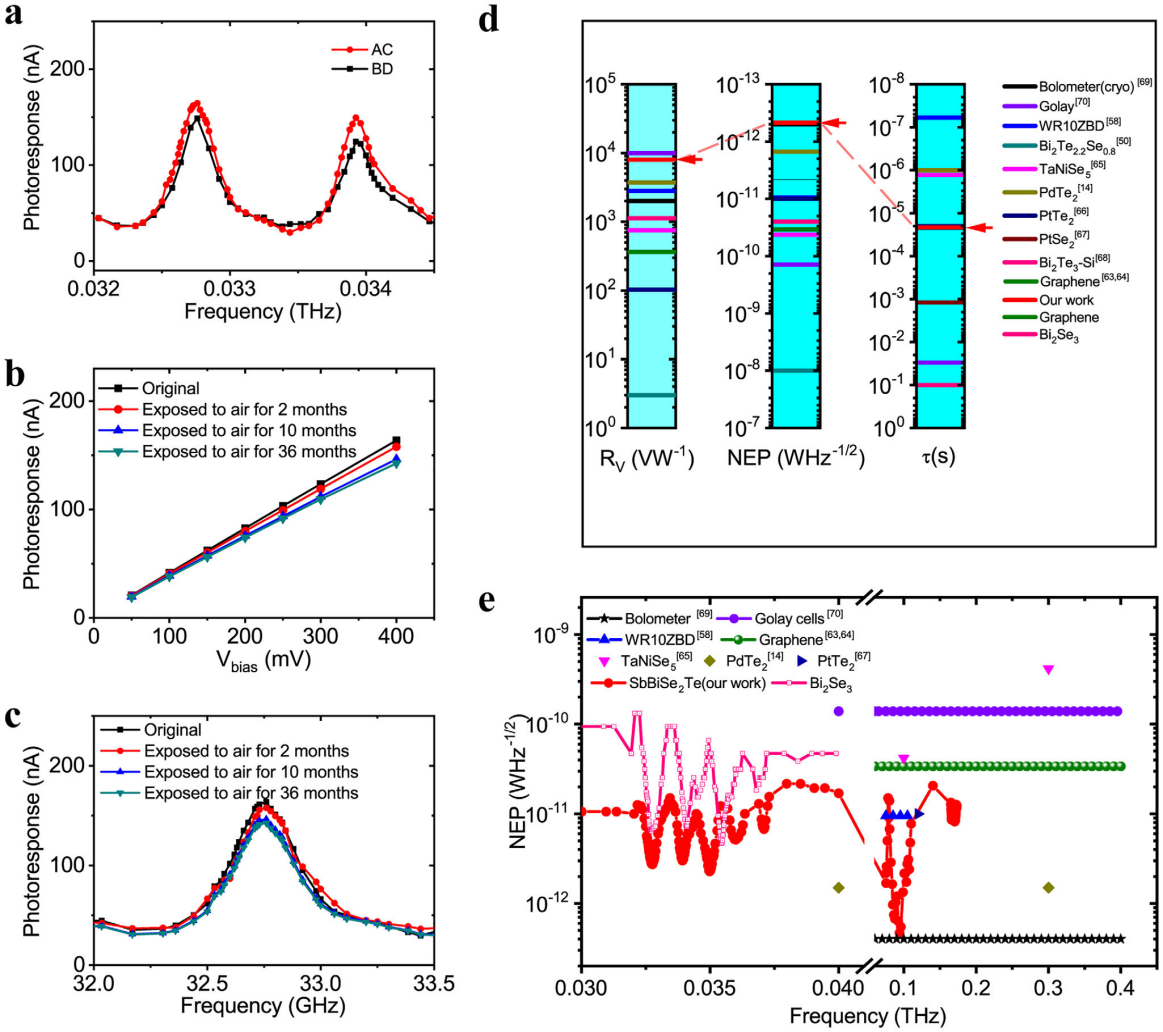
Performance comparison of topological material‐based detectors. (a) The photoresponse comparison between different directions. AC and BD represents the electrodes marked in Figure 2b. (b) Bias‐dependent photoresponse upon exposure to air for 2/10/36 months. (c) Frequency dependent photoresponse upon exposure to air for 2/10/36 months. (d) Comparison of R_V_, NEP, and response speed (τ) of THz/MmW photodetectors based on 2D topological materials, including graphene [63, 64], Bi_2_Te_2.2_Se_0.2_ [50] (zero bias), Ta_2_NiSe_5_ [65]_,_ PdTe_2_ [14] (zero bias), PtTe_2_ [66] (zero bias), PtSe_2_ [67], Bi_2_Te_3_─Si heterojunction [68] (zero bias) and BiSbSe_2_Te. Some typical commercial detectors are also listed. The bolometer needs a Closed‐cycle cryostats, the Golay cells’ modulation frequency is 20 Hz and WRZBD works at 0 bias. A higher location at the coordinate axis represents a better performance for every figure of merit. (e) Frequency‐dependent NEP of some multiband photodetectors based on 2D topological materials.

Our detector demonstrates its equivalent response to different polarization of incident source owing to the rotationally‐symmetric configuration of the couple antenna design (Figure 6a). We also study the stability of the detector by exposing it to air for 2, 10, and 36‐months (Figure 6b,c). The peak photoresponse still perseveres 87% of its initial value after 36 months exposure. The corresponding peak frequency also stay the same position. We summarize R_V_, NEP, response speed, D^*^ and functional frequency range for commercially available low quantum energy detectors and reported 2D topological material‐based detectors (Figure 6d,e; Table S1). The BiSbSe_2_Te‐based photodetector in this work is relatively better compared to other reported 2D topological counterparts. The comparison of working frequency and response speed are also listed, our detectors exhibit a broad spectral band response, however, other technologies usually only perform well for single frequency or narrow band. At room temperature, our detector achieves a competitive performance as commercial bolometer [69] working at cryogenic condition. Compared with Golay cell [70], our detector achieves fast response speed and higher detectivity. Take other 2D topological materials into account, our detector performs a similar response speed, but advances in responsivity and broad spectral band.

In conclusion, topological quaternary BiSbSe_2_Te layers were grown for ultrabroadband photodetection, and the device achieves definitive experimental results of high‐performance, multi‐functional low quantum energy photoresponse. Specifically, the detector exhibits an ultrabroadband spectral response spanning NIR (1064 nm), MWIR (2940 nm) and THz/MmW (9.4, 8.8, and 3.2 mm) regimes, with a room‐temperature responsivity of 8174 V W^−^^1^, noise‐equivalent power of 4.7 × 10^−^^1^ ^3^ W Hz^−^^1^/^2^ and specific detectivity of 1.8 × 10^1^ ^1^ cm Hz^1^/^2^ W^−^^1^ at 0.094 THz under a 400 mV bias. It also demonstrates practical functional features including bias‐tunable spectral response and polarization‐independent photoresponse, endowing it with great potential for diverse low quantum energy optoelectronic applications.

The excellent low‐quantum‐energy photoresponse is hypothesized to be dominated by Dirac plasmons mediated by the intrinsic topological surface states (TSSs) of BiSbSe_2_Te, an inference supported by indirect experimental evidence including bias‐dependent spectral evolution consistent with the Dirac dispersion relation of TSSs and a metallic linear trend of temperature‐dependent resistance. A key limitation is that Drude‐type free‐carrier absorption (surface/bulk) cannot be fully excluded. To achieve a deeper and more complete physical understanding, our follow up work will carry out systematic temperature dependent photoresponse and transport measurements combined with direct spectroscopic and near field characterizations (SNOM, ARPES, THz TDS). In addition, we will further tailor the plasmonic properties by rationally designing and fabricating ordered micro/nanopatterned structures [71], which will enable precise control over the excitation, propagation, and modulation of Dirac plasmons toward optimized device performance.

This work establishes topological BiSbSe_2_Te as a promising material platform and paves a new path for the development of ultrabroadband, high‐sensitivity, multi‐functional and room‐temperature operable low quantum energy photodetectors, providing a valuable design reference for topological material‐based optoelectronic devices targeting the THz and mid‐infrared regimes.

## 4 Experimental Section

### 4.1 Synthesis and Mechanical Exfoliation of BiSbSe_2_Te Crystals

Single crystals of BiSbSe_2_Te were prepared by solid‐state reaction. High‐purity elements Bi (99.999%), Sb (99.999%), Se (99.999%), and Te (99.999%) were weighed in a molar ratio of 1:1:2:1 and loaded into a quartz ampoule. The ampoule was sealed under high vacuum (∼10^−6^. Torr) and heated in a muffle furnace to 800°C over 12 h, held at this temperature for 72 h to ensure complete melting and homogenization, and then slowly cooled to 550°C at a rate of 2°C/h. Afterward, the ampoule was annealed at 550°C for 5 days to improve crystal quality, followed by furnace cooling to room temperature. The resulting ingot was mechanically cleaved for device fabrication

### 4.2 Material Characterizations

The crystal structure and composition investigations were obtained using a scan transmission electron microscope (JEOL 2100F) equipped with an energy dispersive X‐ray spectrometer. Raman measurements were performed using a Nanofinder 30 (TII Tokyo Instruments, Inc) with a 532 nm excitation laser (2 mW). The powder X‐ray diffraction patterns were collected using an X‐ray diffractometer (D/MAX‐2200, Rigaku) with monochromatized Cu‐*Ka* radiation at room temperature in the 2θ range of 10–85° with a scan speed of 10°/min. The components of the crystal were investigated by X‐ray photoelectron spectroscopy (XPS), which was conducted on an XPS scanning microprobe spectrometer (Nexsa, Thermo Fisher) with respect to the position of the C1s peak binding energy at 284.8 eV. The thickness of BiSbSe_2_Te flakes was acquired by an atomic force microscope (Bruker Dimension Edge). The absorption of the thin film was performed by THz time domain measurements (see Figure S3).

### 4.3 THz Time Domain Measurement

For the terahertz time‐domain measurement, a typical transmission terahertz time‐domain spectroscopy system (THz‐TDS) in the frequency range of 0.07–2 THz was used. The size of THz beam at focusing position is about 6 mm. The ultrafast laser system (Coherent Astrella Amplifier) generates 800‐nm, 35‐fs pulses at a 1 kHz repetition rate. The transmission and phase spectra in frequency domain are obtained by Fourier transform of the time‐domain waveforms.

### 4.4 Fabrication and Characterization of the Detectors

The as‐grown crystals, consisting of 1 cm scale single‐crystalline block, were successively mechanically exfoliated on a silicon wafer with an insulating 280 nm thick SiO_2_ top‐layer. The detectors were fabricated by ultraviolet lithography, and Ti/Au (10/100 nm) contact electrodes were deposited using electron‐beam evaporation. For photocurrent measurements, the light source with the wavelength of 1064 and 2940 nm were generated from solid‐state lasers and calibrated by an optical power meter. THz wave sources were based VDI tenable synthesizer (0.008–0.02 THz). For such VDI tenable synthesizer, the frequency was tuned with Signal Generator Extension Module WR28SGX (0.026–0.040 THz), Mini Signal Generator Extension Module WR10SGX (0.075–0.110 Hz) and 165–173 GHz Modular Tx304 Multiplier with Multiplier Output 175 × 2. The output radiation was calibrated by a thermal Golay cell. The photoresponse is recorded by a lock‐in amplifier after a low‐noise current preamplifier. The amplifier converts photocurrent into a voltage output, and the responsivity is reported in units of mV/A. To evaluate performance of the detector, several Figures of merit are used, such as photoresponsivity (*R_V_*), NEP, and D^*^. They are defined as [45]

(1) $$R_{V}=\frac{V}{pA};NEP=\frac{v_{n}}{R_{V}};D^{*}=\frac{\sqrt{A}}{NEP}$$

where V is the photovoltage of the detector, p is the power density calibrated by a Golay cell (the absorption area of the Golay cell is 50 mm^2^, and responsivity of the Golay cell is 105 V W^−1^ at 15 Hz) and it has a typical value of 0.00143Wm^−2^ at 0.094 THz. A is the effective absorption area of the detector, described as A = Gλ^2^/(4π) [45] if assuming the antenna is matched to its load (G is the gain of the antenna and G = 1.44 calculated by HFSS(see Figure S11), lambda is the wavelength of the incident light). *v_n_* is the measured noise voltage.

### 4.5 Estimation of Bolometric Effect

The influence of bolometric effect can be estimated under the electromagnetic radiation. The temperature relaxation of the bolometric sensitive surface is described by the following heat balance equation:

$$C\frac{d\Delta T}{dt}+G\Delta T=\eta \;Pe^{i\omega t}$$

where C is the specific heat, G is the thermal conduction, ΔT is the temperature difference, P is the incident power, η is the absorption, ω is the modulation frequency. Then ΔT can be achieved by solving the equation:

$$\Delta T=\Delta T_{0}\;e^{-\frac{Gt}{C}}+\frac{\eta Pe^{i\omega t}}{G+i\omega C}$$

$$\Delta T=\frac{\eta P}{\sqrt{\omega^{2}C^{2}+\frac{C^{2}}{\tau^{2}}}}$$

where $\tau =\frac{C}{G}\;$ refers to the response time of the detector and can be obtained from the amplitude‐frequency characteristics of the detector. Then the maximum responsivity caused by bolometric effect at room temperature can be calculated by:

$$\begin{array}{ccc} \;R_{V} & = & \frac{\Delta U}{P}=\frac{\Delta RU_{bias}}{RP}=\frac{\alpha \eta \Delta TU_{bias}}{P}=\frac{\alpha \eta U_{bias}}{\sqrt{\omega^{2}C^{2}+\frac{C^{2}}{\tau^{2}}}}\\ & & =\frac{\alpha \eta U_{bias}}{\sqrt{\omega^{2}m^{2}C_{m}^{2}+\frac{m^{2}C_{m}^{2}}{\tau^{2}}}} \end{array}$$

where the thermal coefficient of resistance (TCR) $\alpha =\frac{\Delta R}{R\Delta T}=0.004\;K^{-1}$ can be directly derived from experiments (Figure S5b). For the specific heat per unit mass *C_m_* and absorption in THz wave range, we can use the data from Bi_2_Te_3_ to do the estimation. As reported [72, 73], the absorption coefficient is ∼400 cm^−1^, therefore, for our nanosheet with thickness of 98 nm, the absorption is only about 3.9 × 10^−3^. The specific heat per unit mass *C_m_* is about 0.20 *Jg* ^−1^ *K* ^−1^. Also, in our case, *U_bias_* =  0.4 *V*,  *C*  = *mC_m_* ,*m*  =  ρ*V*, ρ  =  6.4 *gcm* ^−3^,*V*  =  *abc*,  *a*  =  *b*  =  5 × 10^−5^ *m*, *c*  =  1 × 10^−7^ *m*. For THz response, τ  =  21.7 µ*s*,  ω  =  1*kHz*, Then *R_V_* =  0.077 *VW* ^−1^, which is much smaller than the measured value for terahertz response.

## Author Contributions

J.C.T. supervised this work. P.Y. supervised the material growth. J.C.T. and T.N.Z conceived the idea. J.C.T and T.N.Z proposed the model and did the experimental work. J.T.Z. and T.T.H. synthesized the material and did the characterization. L.Y. and L.J.Z did the first‐principle calculation. H.L. did the numerical simulation. B.Y.Y., J.H.R., X.C., X.C., N.D., J.L.W., and J.H.C. discussed the results. J.C.T. and T.N.Z. wrote the manuscript.

## Funding

This paper was supported by the National Key Research and Development Program of China (Grant No. 2024YFB4207100), Science and Technology Commission of Shanghai Municipal Government (Grant No. 25DP1500600), Science and Education Integration Project from Shanghai Institute of Technical Physics (grant no. SITPKJRH‐2025‐01), the National Natural Science Foundation of China (Grant No. 22175203), and the Natural Science Foundation of Guangdong Province (Grant No. 2022B1515020065).

## Conflicts of Interest

The authors declare no conflicts of interest.

## Supporting information




**Supporting File**: advs74725‐sup‐0001‐SuppMat.docx

## Data Availability

The data that support the findings of this study are available from the corresponding author upon reasonable request.
