# Supplementary material for: Dirac Surface‐State Driven Broad Spectral Band Low Quantum Energy Photoresponse in Quaternary Topological BiSbSe2Te
Source: Adv Sci (Weinh). 2026 Mar 12;13(28):e22592. doi: 10.1002/advs.202522592 (PMC13185879; doi:10.1002/advs.202522592)
Supplement: Supplementary file 1 — Supporting File: advs74725‐sup‐0001‐SuppMat.docx [file ADVS-13-e22592-s001.docx]

Supporting Information for

**Dirac Surface-State Driven Broad Spectral Band Low Quantum Energy Photoresponse in** **Quaternary Topological BiSbSe_2_Te**

**This file includes:**

**Figs. S1 to S11**

**Note S1 to S2**

**Tables S1**

**References**

**1. X-ray diffraction (XRD) and energy dispersive spectra (EDS) characterization of BiSbSe_2_Te**

We first confirm the formation of a single phase by carrying out X-ray diffraction measurements using an X-ray diffractometer (D/MAX-2200, Rigaku) with monochromatized Cu-*Ka* radiation. The (006), (015), (1010), (0015), (0210), (0120) as well as (125) peaks can be clearly identified. The measured X-ray powder diffraction patterns of as-obtained BiSbSe_2_Te are shown in **Fig. 1a**. EDS is also used for the element analysis of materials. The energy dispersive X-ray spectroscope equipped on TEM shows the characteristic peaks of Bi, Sb, Se, and Te elements, while the energy dispersive X-ray spectroscope equipped on SEM results in Bi_1.09_Sb_0.91_Se_2_Te (**Fig. S1c, d**), which is close to the stoichiometric ratio of 1:1:2:1.


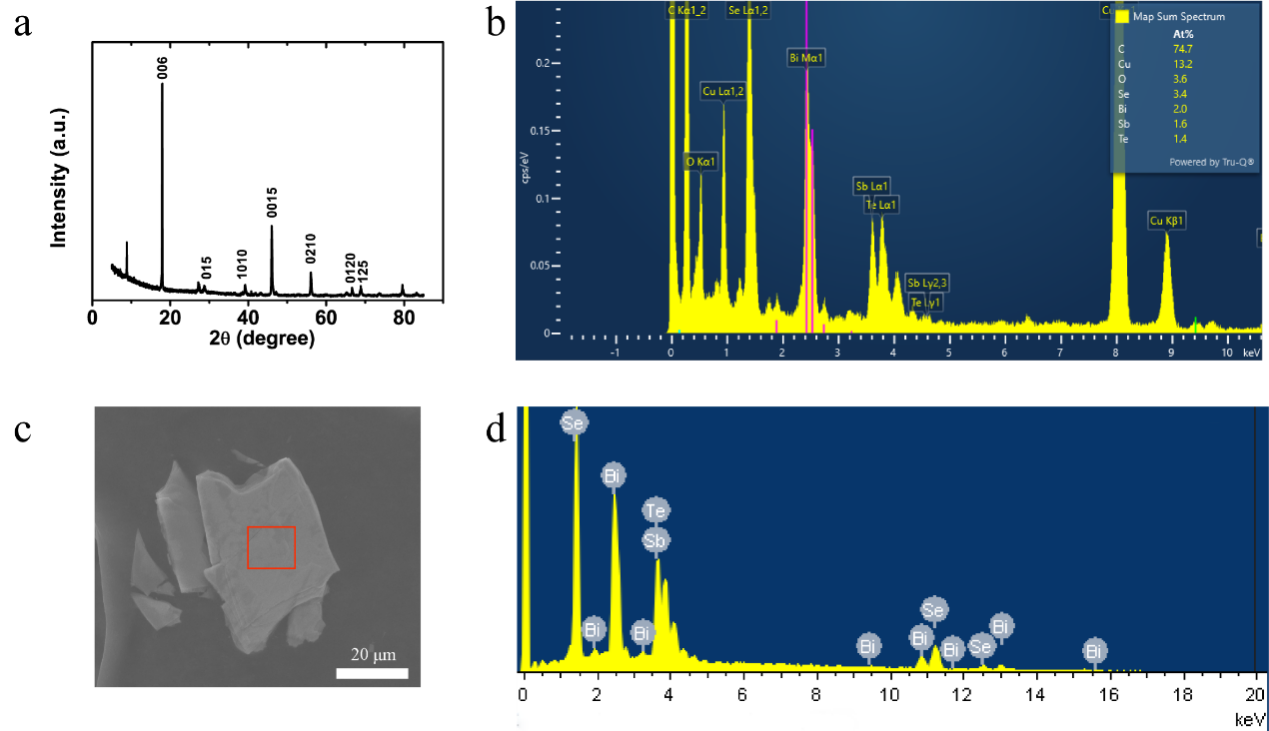


**Fig. S1. Structure and characterization of** BiSbSe_2_Te**.** **a**, X-ray powder diffraction (XRD) patterns for BiSbSe_2_Te crystals. **b**, EDX spectrum of a BiSbSe_2_Te flake. **c, d,** Scan electron microscopy (SEM) image and corresponding energy dispersive X-ray spectrum of a BiSbSe_2_Te flake. The red rectangle represents the measured area. The EDS results show the atomic ratio of Bi : Sb : Se : Te in crystals is 1.09 : 0.91 : 2.0 : 1.

**2. X-ray photoelectron spectroscopy (XPS) characterization of BiSbSe_2_Te**

The chemical analysis was also made by XPS (**Fig. S2**). The XPS experiment was performed on an XPS scanning microprobe spectrometer (Nexsa, Thermo Fisher). The sample was calibrated by the carbon peak located at 284.8 eV. The energy resolution was approximately 0.05 eV. The calculated XPS results show the atomic ratio of Bi : Sb : Se : Te in crystals is 1.2 : 1.01 : 1.79 : 1.


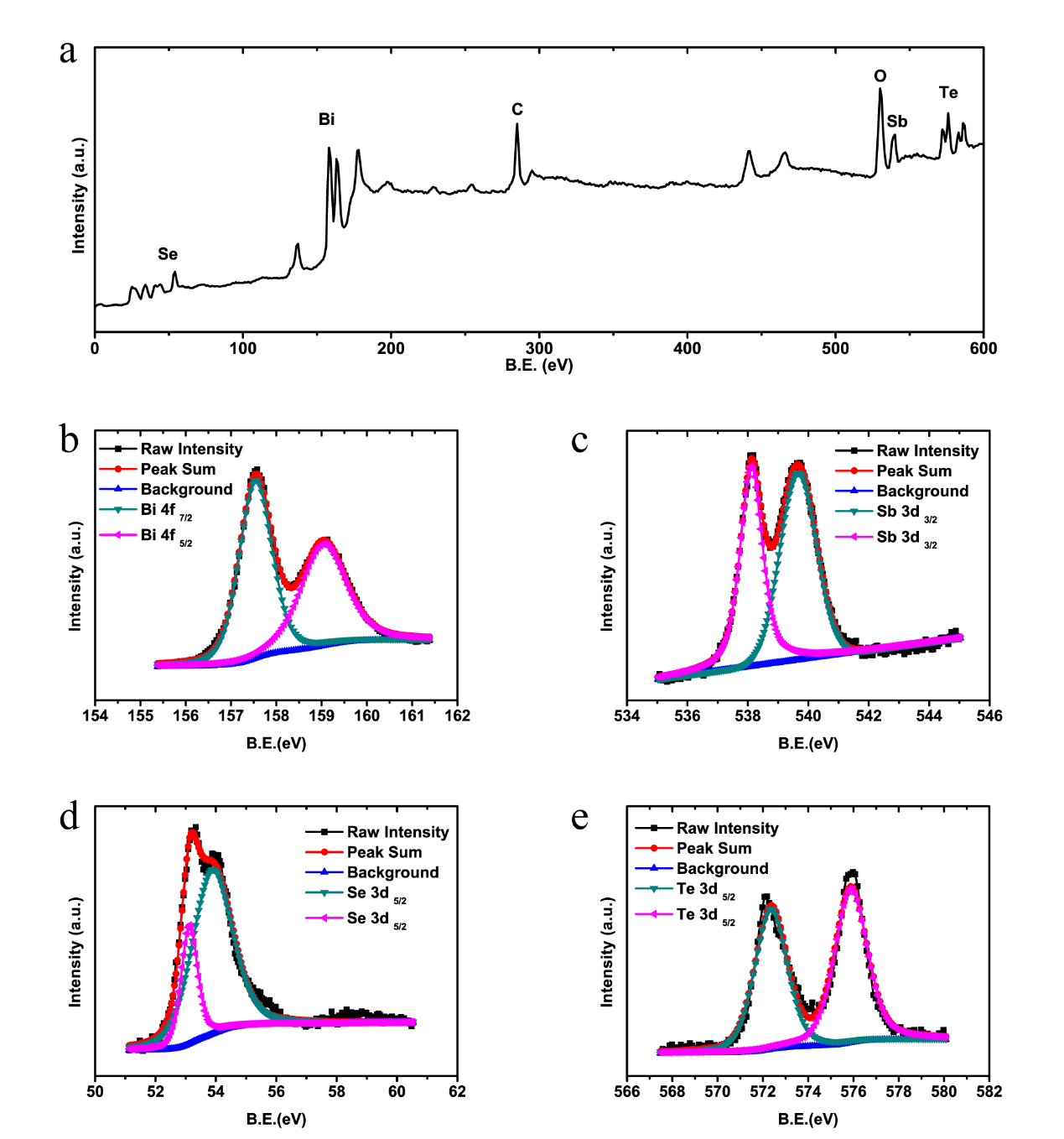


**Fig. S2. XPS characterization of BiSbSe_2_Te.** **a**, XPS spectrum ofBiSbSe_2_Te. **b, c,** **d, e,** High-resolution XPS spectra of the Bi 4f, Sb 3d Se 3d and Te3d regions. The XPS results show the atomic ratio of Bi : Sb : Se : Te in crystals is 1.2 : 1.01 : 1.79 : 1.

**3. THz time domain measurement**

To verify the absorption of BiSbSe_2_Te in THz range, we measure the THz transmission spectrum of this BiSbSe_2_Te with antenna and substrate. As show in **Fig. S3**, it exhibits obvious absorption in a broad spectral band range. The substrate absorption is also shown as a contrast.


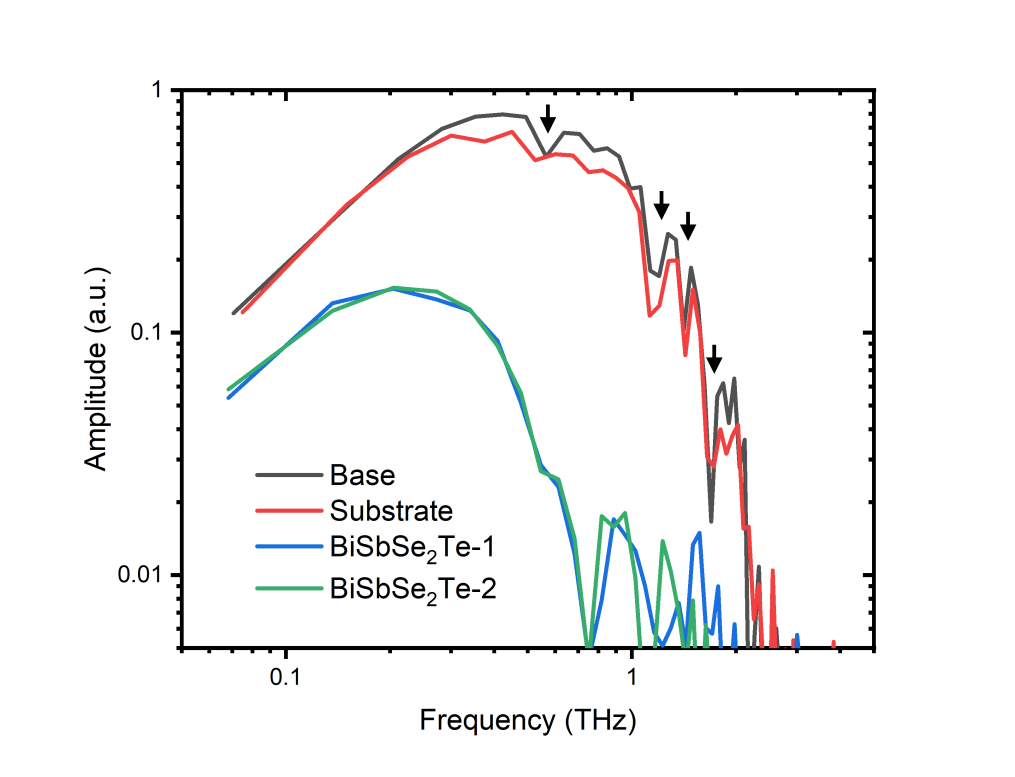


**Fig. S3.**  Terahertz time-domain transmission spectrum. two samples together with substrate are compared. The black arrows indicate the absorption of H_2_O and O_2_ in air. They are not eliminated to verify the sensitivity of the spectrum.

**4. Detector design**

In our design, the antenna parameter of (**Fig. S4**) **a, d1, d2, L** equals to 150 μm, 2.5 μm, 20 μm and 759 μm, separately. The thickness of the BiSbSe_2_Te flake is 98 nm and the thickness of the dielectric SiO_2_ layer is 280 nm.


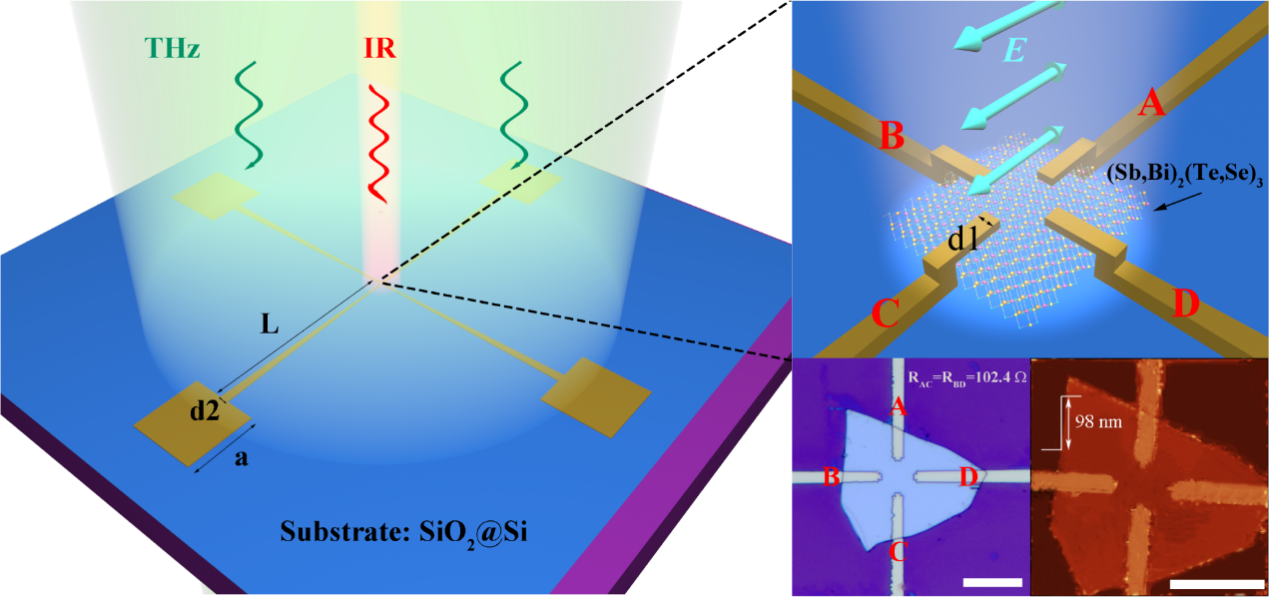


**Fig. S4.** Schematic diagram of the detector structure. L is the length of the antenna. d1 and d2 represent length of tip side and bottom side of the antenna. ***E*** denotes the TM polarization orientation (to arouse SPPs) of incident electromagnetic wave. Two inset Figures show the optical and Atomic Force Microscope (AFM) scan of the detector. Both of the scale bar is 20 μm.

**5. I-V characteristic of BiSbSe_2_Te detector**

The good linearity and symmetry of the I-V characteristic curve, shown in **Fig. S5**, demonstrates good ohmic contacts in the detector. The temperature-dependent resistance of the detector is measured from 77-340 K. The Temperature-dependent resistance of the BiSbSe_2_Te film is also shown in **Fig.** **S5b,** the resistance follows the metallic linear trend, which is the same as Te doped Bi_2_Se_3_^[6]^


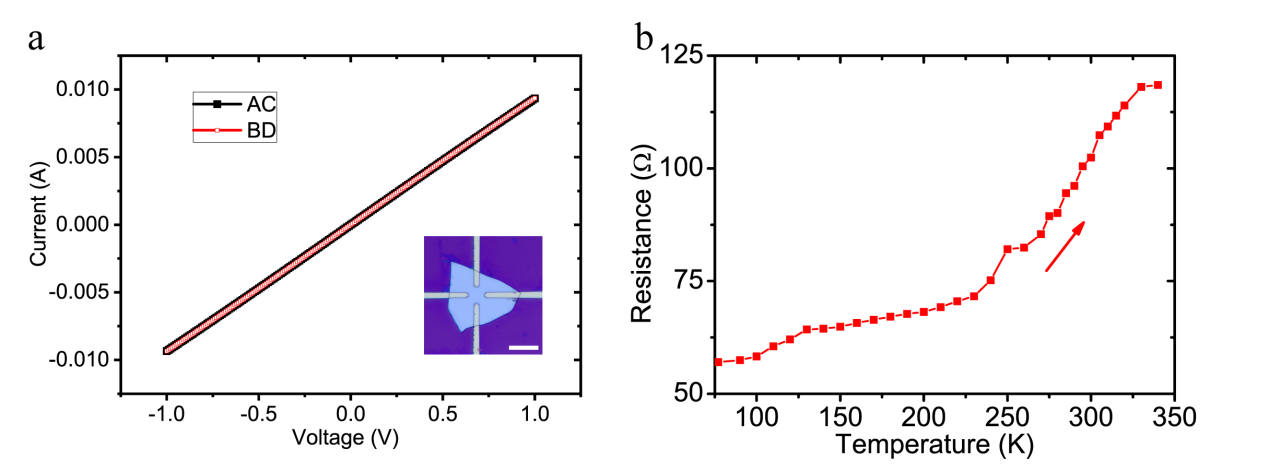


**Fig. S5. Electrical characterization. a,** I-V curve of the detector. AC and BD represents the electrodes along different directions marked in **Fig. S4.** The scale bar in the insert figure is 20 μm. **b,** Temperature-dependent resistance of the BiSbSe_2_Te film.

**6. Measured Noise and Time-resolved photocurrent responses**

We measured the noise of the detector at different bias (**Fig. S6a**). At low frequencies, 1/f noise dominates, which originates from fluctuations of local electronic states induced by the disorder or defects. However, at higher frequencies, the 1/f noise decays quickly while the thermal Johnson–Nyquist noise ($v_{t}$) and the dark current shot noise ($v_{b}$) dominate. Then the measured noise hardly varies with frequency. At a frequency of 10^4^ Hz and a bias of 400 mV, the measured noise level is 3.84×10^−9^ VHz^-1/2^.

the accurate response speed of 21.76/20.1 μs (rise/fall time) at 0.34 THz was captured directly from the oscilloscope. The THz sourse was modulated by the signal generator with a frequency of 1500 Hz.

Multiple devices were characterized to evaluate random device-to-device variation and confirm that the observed photoresponse is reproducible and statistically reliable, rather than originating from accidental device-specific artifacts. What’s more, these devices reveal no obvious correlation between the device responsivity and the thickness variation. The detectivity will increase slightly with increasing in thickness.


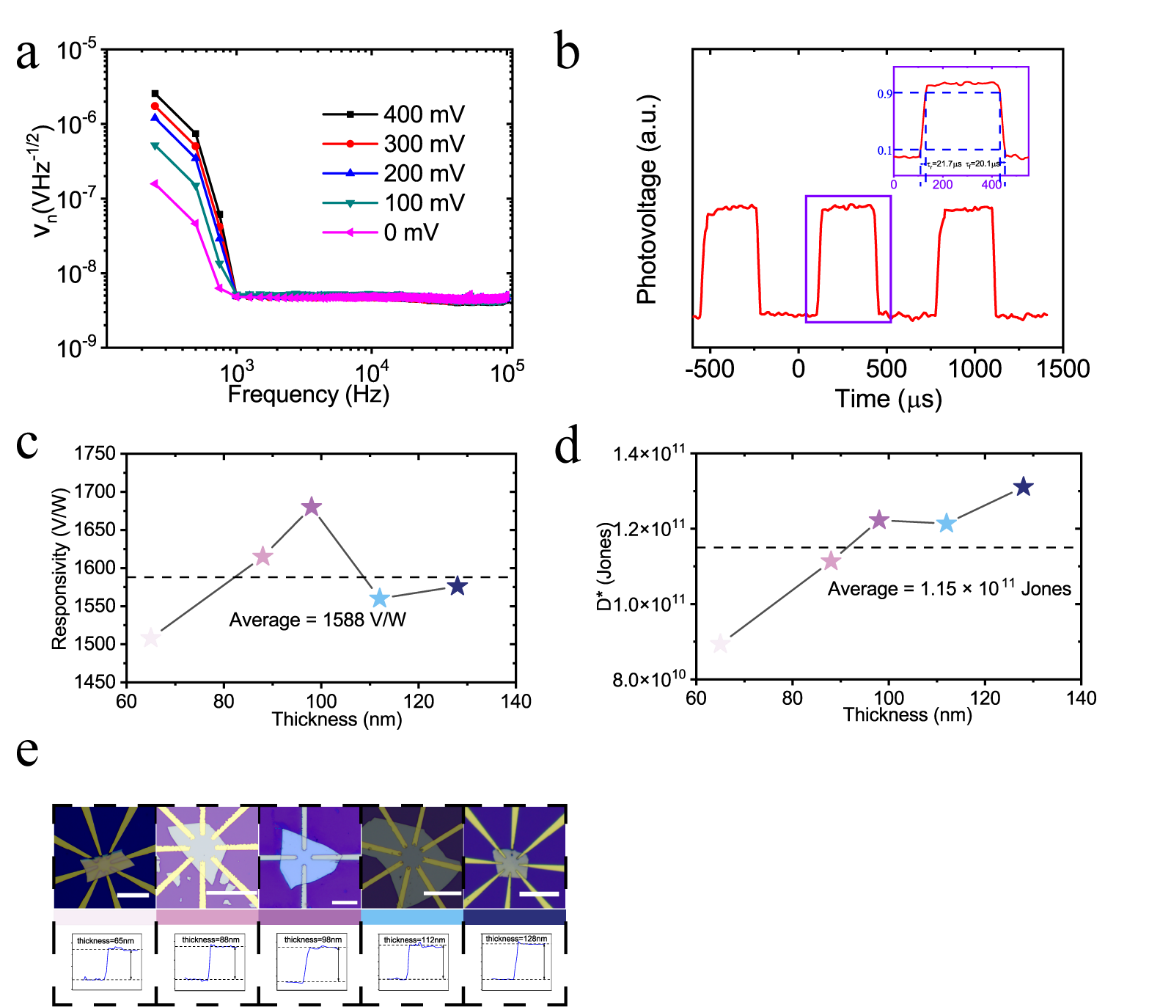


**Fig. S6.a** The voltage noise spectra at different bias of BiSbSe_2_Te photodetector, **b**. Photoresponse speed of the photodetector under 0.034 THz. c, d, e, R_V_ and D* of 5 typical BiSbSe_2_Te devices at 0.034 GHz (peak value) and their related optical microscope images and thickness achieved by AFM. The scale bar is 20 micrometers.

**7. Band structure calculations**

Within the density functional theory, the first-principle calculations were performed with the Vienna ab initio simulation package (VASP)^[1]^ (**Fig. S7**), which utilized the generalized gradient approximation (GGA)^[2]^ in the Perdew-Burke-Ernzerhof (PBE) exchange-correlation functionals^[3]^. The lattice parameters were optimized to be a = b = 4.3 Å, c = 30.4 Å, which are accordance with the experimental values. The plane-wave cutoff energy was set to be 550 eV, and a Γ-central 16 × 16 × 2 k-point was used in our calculations. The spin-orbit coupling (SOC) effect was also considered in our band structure calculations. The convergence criteria for energy and atom forces were set be 105 eV and 0.01 eV/Å, respectively. To investigate the projected surface states, a first-principles tight-binding model Hamiltonian was firstly built by the maximally localized Wannier function (MLWF) method^[4]^, where the p orbitals of Bi, Sb, Se and Te atoms were chosen as basis. Then, the Wannier Tools package was used to investigate the surface states in the framework of the constructed Hamiltonian^[5]^.


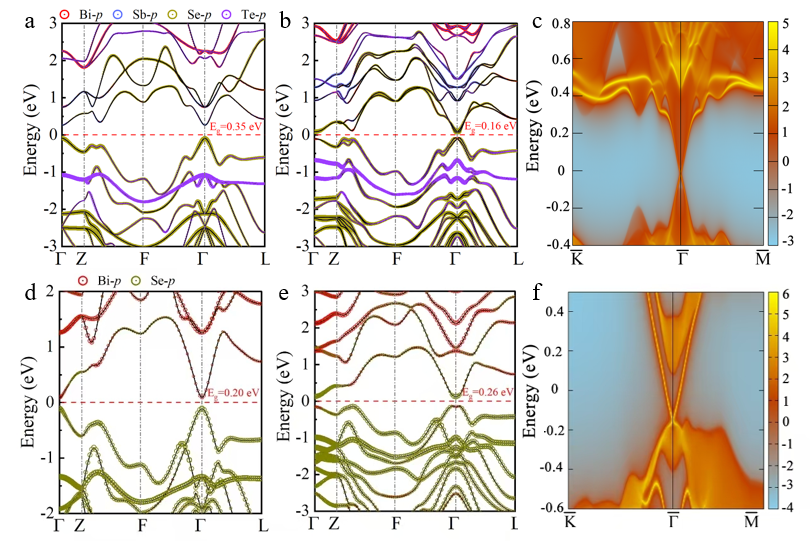


**Fig. S7. Calculated band structure of BiSbSe_2_Te and Bi_2_Se_3_. a, d** band structure of BiSbSe_2_Te and Bi_2_Se_3_ without inclusion of SOC effects; **b, d** band structure of BiSbSe_2_Te and Bi_2_Se_3_ with inclusion of SOC effects. The contributions of the p orbitals of Bi, Sb, Se and Te atoms are colored. **c, f** Calculated surface band structure of BiSbSe_2_Te and Bi_2_Se_3_, respectively.

**8. The performance of** **Bi_2_Se_3_ detector**

To study the effect of Sb dopant on the performance, we fabricate a Bi_2_Se_3_ based detectors with the same antenna in **Fig.** **S4a** for comparation. The performance of Bi_2_Se_3_ detector is shown in **Fig.** **S8abc**. The Bi_2_Se_3_ detector exhibited similar patterns to the BiSbSe_2_Te detectors, but its performance was significantly inferior to that of the BiSbSe_2_Te detector. The peak R_v_ of Bi_2_Se_3_ detector is 810 V/W, which is smller than that of BiSbSe_2_Te detectors (1588 V/W). The peak D^*^ (4.25 ×10^10^ Jones).

34


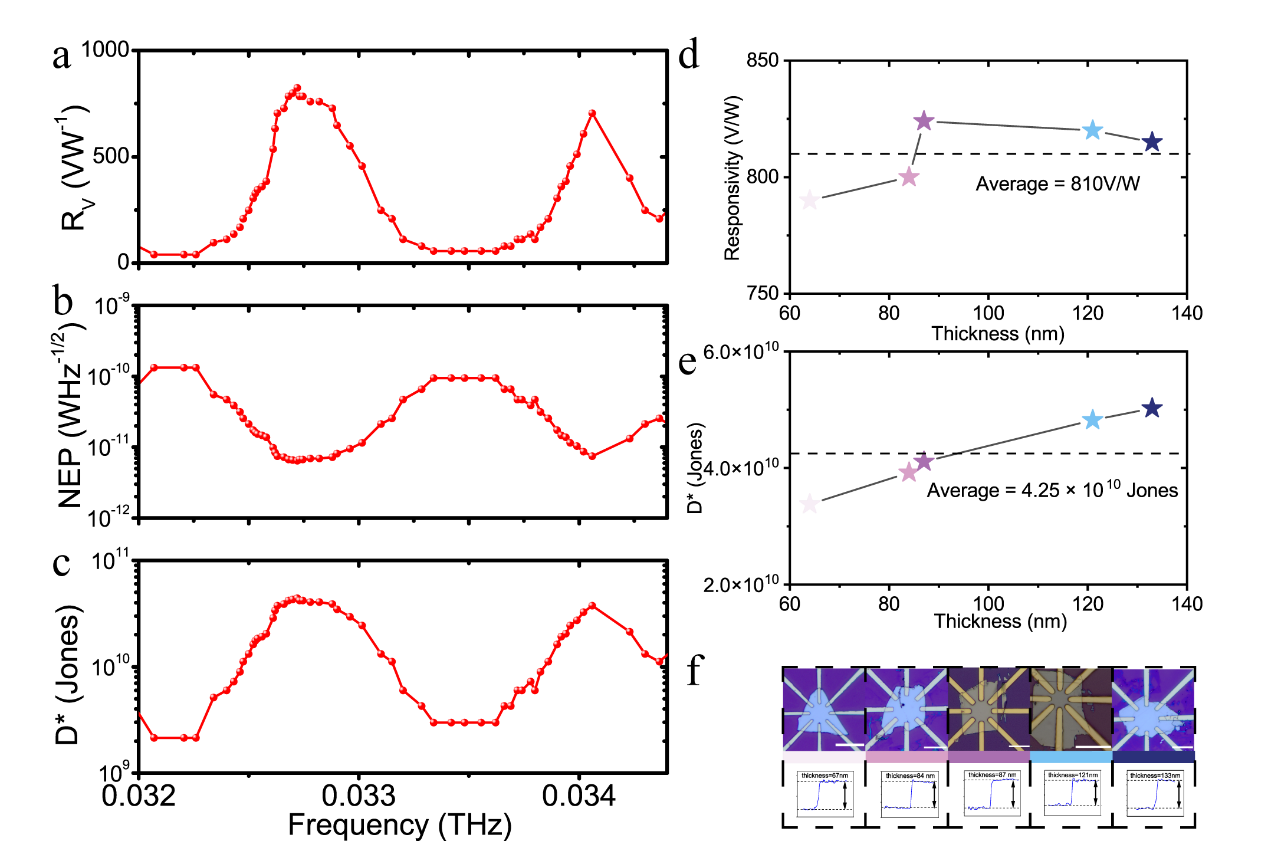


**Fig. S8.** **a,b,c** R_V_, NEP, and D* of the Bi_2_Se_3_ detector in a frequency range of 0.032–0.0345 THz under a voltage bias of 400 mV. **d,e,f** R_V_ and D* of 5 typical devices at 0.0327 GHz (peak value) and their related optical microscope images and thickness achieved by AFM. The scale bar is 20 micrometers.

**9. The performance for 0.168 THz radiation in a detector with a bow-tie antenna**

The dipole-like detector exhibits a broadband spectral response across the 0.032 to 0.173 THz range but shows suboptimal performance at 0.168 THz. To optimize the performance at 0.168 THz, we fabricate a detector with bow-tie antenna which is designed for 0.68 THz radiation. The space between two electrodes is 10 μm. the marked parameters (**Fig. S9a**) **L, α, R** equal to 100 μm, 90°, and 200 μm, separately. R_V_, NEP, and D* of the detector at different voltage bias for room temperature under incident sources of 0.168 THz are shown in **Fig. S9c-e**.


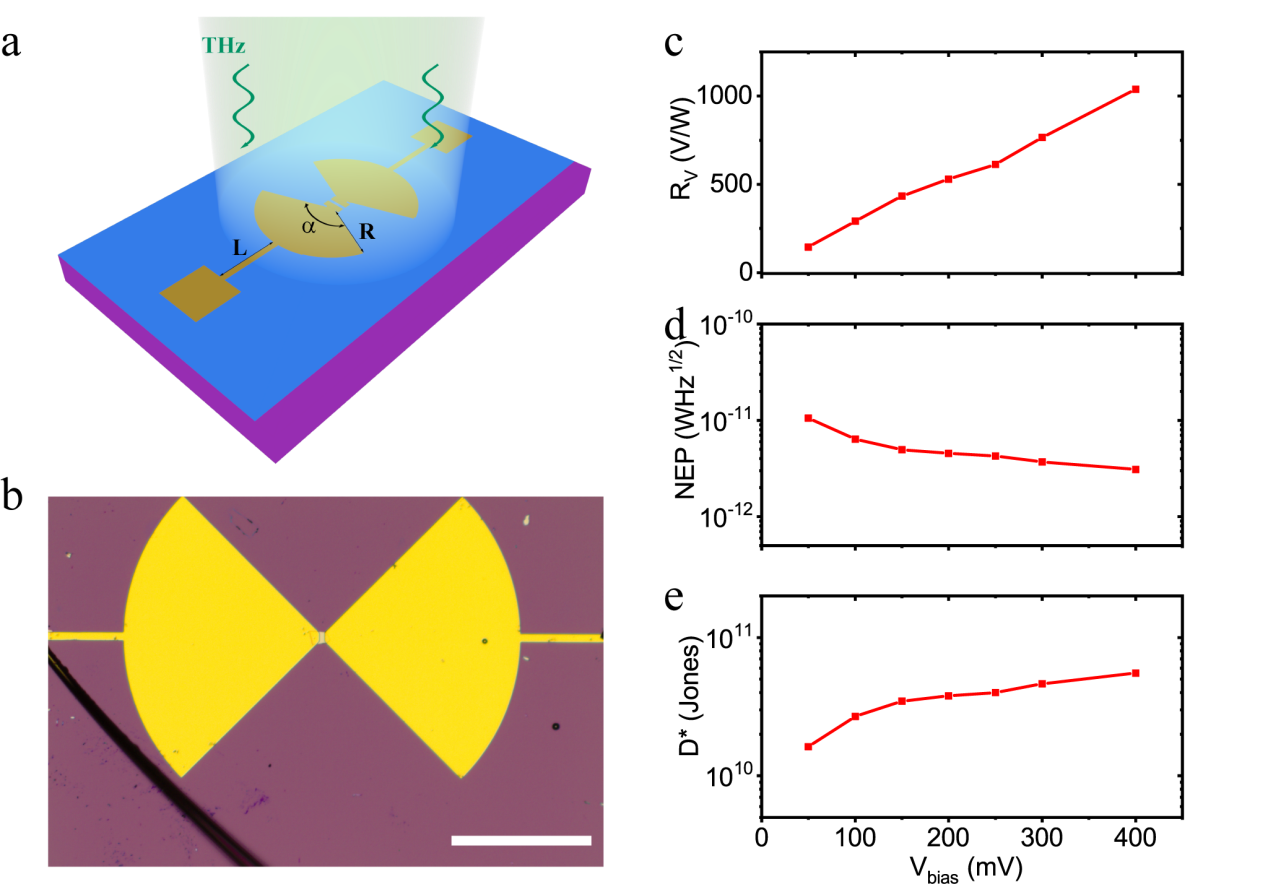


**Fig. S9. Structure and characterization of** **BiSbSe_2_Te.** **a**, Schematic of the bow-tie antenna-assisted detector. **b,** optical image of the detector. the scale bar is 100 μm **c, d, e,** R_V_, NEP, and D* of the detector at different voltage bias for room temperature under incident source of 0.168 THz.

**Supporting Note S1**

To estimate the resonant plasma frequency of the BiSbSe2Te material, we conduct Hall measurement. Based on the measured carrier density (on the order of 10^22^cm^-3^) and effective mass, we used the Drude model to calculate the plasmon resonance frequency:

$$\omega_{p}=\sqrt{\frac{ne^{2}}{\varepsilon_{0}\varepsilon_{r}m^{*}}} f_{p}=\frac{\omega_{p}}{2\pi}$$

Where ω_p_ is the plasma angular frequency, n is the free carrier concentration and equals to 5.95×10^22^ cm^-3^ (**Fig.10f**), *e* is the elementary charge and equals to 1.602×10^−19^ C, ε_0_ is the vacuum permittivity and equals to 8.854×10^−12^ F/m, ε_r_ is the ε_0_ the environmental dielectric constant and equals to 2.5, considering the Si/SiO_2_ substrate. m^*^ is the effective mass of the charge carriers and is approximately one tenth of the mass of electrons in Quaternary Topological BiSbSeTe, that is, 9.1×10^−32^ kg.

Then the plasmon resonance frequency equals to 4.38 ×10^14^ Hz.


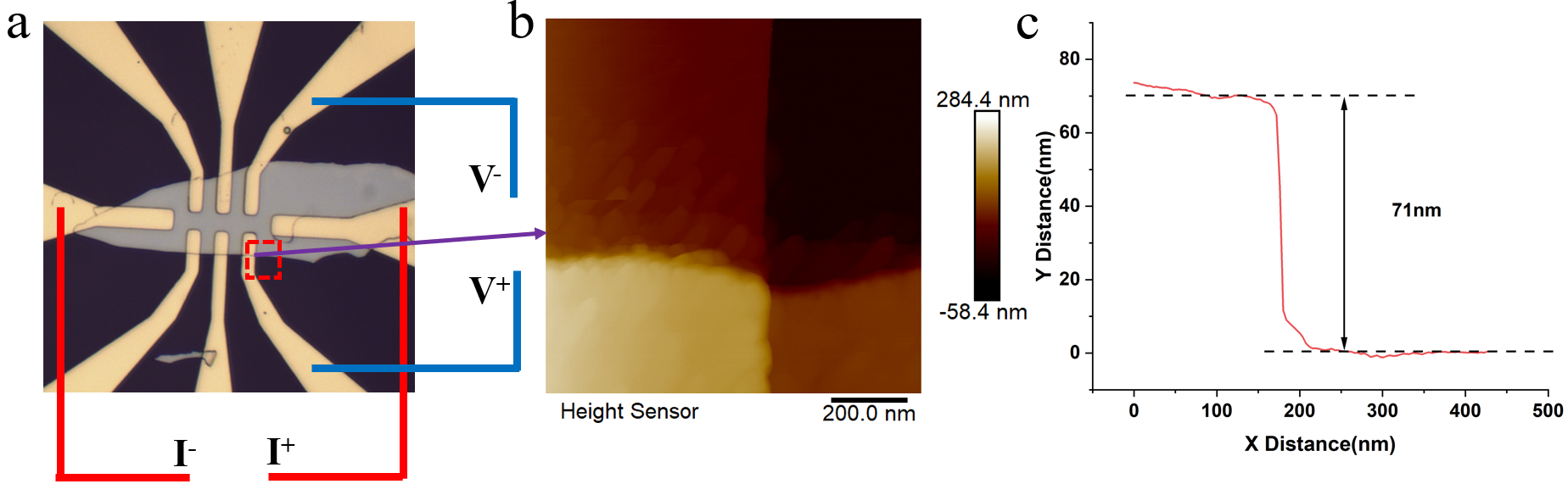

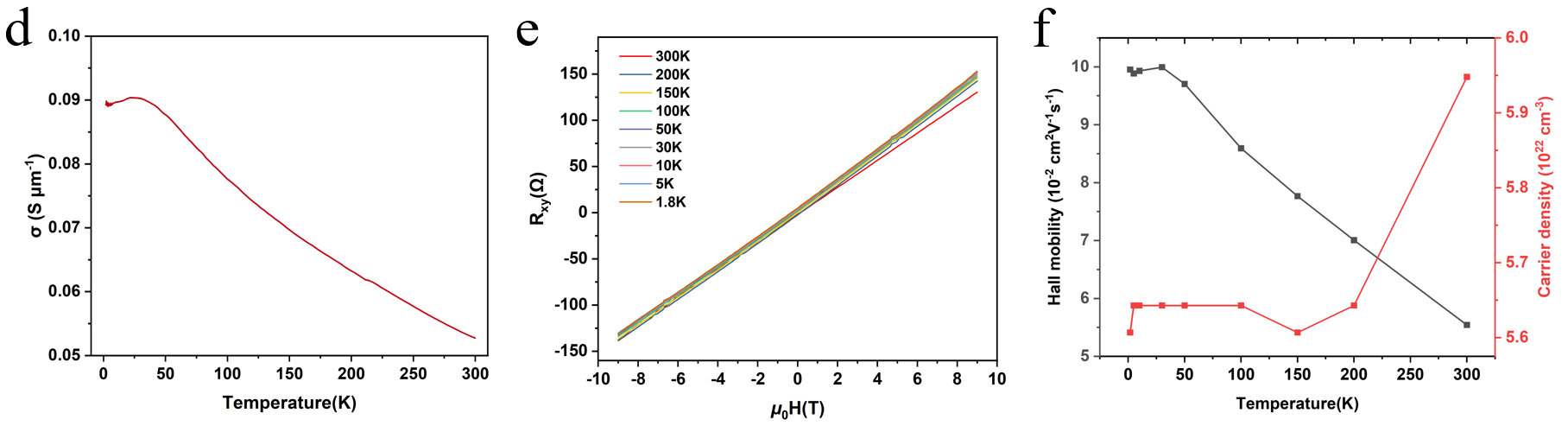


**Fig. S10.** **Hall measurement of BiSbSe_2_Te.** **a**, optical image of the Hall measurement and **b, c,** its related AFM test results. **d, e,** **f**, Hall results from 1.8K to room temperature.

**Supporting Note S2**

To estimate the gain of our designed antenna, High Frequency Structure Simulator (HFSS) software is used to simulate this antenna and the highest gain is 1.44 as shown in Fig.S11b.


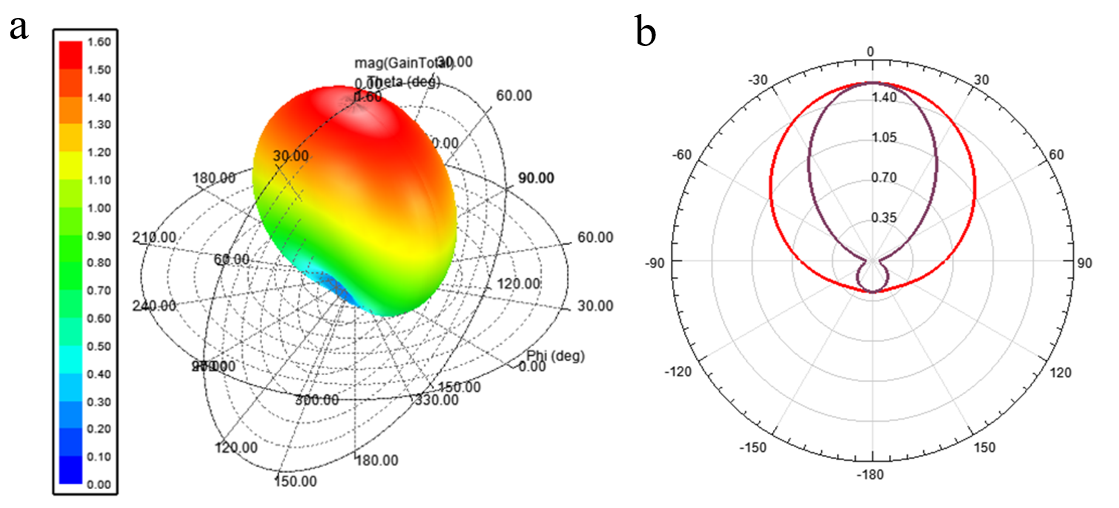


**Fig. S11. simulation of antenna at 0.075 THz.** **a**, 3D polar plot and **b,** its related radiation patten. The red curve and the purple curve respectively represent the cases where the phi Angle is 0 and 90 degrees.

**Table S1** Detector parameters for detectors based on 2D topological materials and a few references for state-of-the-art detectors not based on 2D materials.

| type | R_v_ (VW^-1^) | NEP (WHz^-1/2^) | Response time | D^*^ (Jones) | F (THz) | Ref. |
| --- | --- | --- | --- | --- | --- | --- |
| Bolometer (4K) | 2000 | 5×10^-13^ | 0.05 ns | - | 0.3-3 | ^[8]^ |
| Golay (Commercial) | 10000 | 1.4×10^-10^ | ~30 ms | 7×10^9^ | 0.02-20 | ^[9]^ |
| Schottky diodes (WR10ZBD) | 2800 | 9.5×10^-12^ | 0.06 μs | - | 0.075-0.11 | ^[10]^ |
| Bi_2_Se_3_ and  Bi_2_Te_2.2_Se_0.8_ | 3 and 0.21 | 1×10^-8^ | - | - | 0.2927 and 0.3326 | ^[6]^ |
| TaNiSe_5_ | 755 VW^-1^ | 4.2×10^-11^ | 1.1 μs |  | 0.1, 0.3 | ^[11]^ |
| PdTe_2_ | 3720 | 1.5×10^-12^ | 1 μs | - | 0.04, 0.12, 0.3 | ^[12]^ |
| PtTe_2_ | 102.4 | 1×10^-11^ | 20 μs | - | 0.12 | ^[13]^ |
| PtSe_2_ | 4.5 AW^-1^ | - | 1.2 ms | 7×10^8^ | 632-10000 nm | ^[14]^ |
| Bi_2_Te_3_-Si(broadband) | 1 AW^-1^ | 2.5×10^-11^ | 100 ms | 7.5×10^5^ | 370 nm-118 μm | ^[15]^ |
| Graphene | 764 | 3.4×10^-11^ | - | - | 0.05-0.45 | ^[16]^ |
| BiSbSe_2_Te | 8000  1600 | 4.7×10^-13^  2.29×10^-12^ | 21.7 μs | 1.8×10^11^  1.23×10^10^ | 0.03-0.04  0.075-0.11  0.168 | Our work |

**References**

[1] G. Kresse, J. Furthmüller, *Phys. Rev. B* **1996**, 54, 11169.

[2] a) P. E. Blöchl, *Phys. Rev. B* **1994**, 50, 17953; b) P. E. Blöchl, O. Jepsen, O. K. Andersen, *Phys. Rev. B* **1994**, 49, 16223.

[3] J. P. Perdew, K. Burke, M. Ernzerhof, *Phys. Rev. Lett.* **1996**, 77, 3865.

[4] I. Souza, N. Marzari, D. Vanderbilt, *Phys. Rev. B* **2001**, 65, 035109.

[5] Q. Wu, S. Zhang, H.-F. Song, M. Troyer, A. A. Soluyanov, *Comput. Phys. Commun.* **2018**, 224, 405.

[6] L. Viti, D. Coquillat, A. Politano, K. A. Kokh, Z. S. Aliev, M. B. Babanly, O. E. Tereshchenko, W. Knap, E. V. Chulkov, M. S. Vitiello, *Nano Lett.* **2016**, 16, 80.

[7] a) S. Nimanpure, A. Pandey, G. Singh, B. P. Singh, D. R. Chowdhury, Y. U. Jeong, R. Sharma, S. Husale, M. Jewariya, *Optical Materials* **2021**, 121, 111490; b) N. P. Gorbachuk, V. R. Sidorko, *Powder Metallurgy and Metal Ceramics* **2004**, 43, 284.

[8] Bolometer <https://www.scontel.ru/terahertz/>, accessed.

[9] Golay <http://www.tydexoptics.com/products/thz_devices/golay_cell/>, accessed.

[10] Schottky diodes <https://www.vadiodes.com/en/products/detectors>, accessed.

[11] Z. Dong, W. Guo, L. Zhang, Y. Zhang, J. Chen, L. Huang, C. Chen, L. Yang, Z. Ren, J. Zhang, W. Yu, J. Li, L. Wang, K. Zhang, *Advanced Science* **2022**, 9, 2204580.

[12] C. Guo, Y. Hu, G. Chen, D. Wei, L. Zhang, Z. Chen, W. Guo, H. Xu, C.-N. Kuo, C. S. Lue, X. Bo, X. Wan, L. Wang, A. Politano, X. Chen, W. Lu, *Sci. Adv.* **2020**, 6, eabb6500.

[13] H. Xu, C. Guo, J. Zhang, W. Guo, C.-N. Kuo, C. S. Lue, W. Hu, L. Wang, G. Chen, A. Politano, X. Chen, W. Lu, *Small* **2019**, 15, 1903362.

[14] X. Yu, P. Yu, D. Wu, B. Singh, Q. Zeng, H. Lin, W. Zhou, J. Lin, K. Suenaga, Z. Liu, Q. J. Wang, *Nat. Commun.* **2018**, 9, 1545.

[15] J. Yao, J. Shao, Y. Wang, Z. Zhao, G. Yang, *Nanoscale* **2015**, 7, 12535.

[16] a) S. Castilla, B. Terrés, M. Autore, L. Viti, J. Li, A. Y. Nikitin, I. Vangelidis, K. Watanabe, T. Taniguchi, E. Lidorikis, M. S. Vitiello, R. Hillenbrand, K.-J. Tielrooij, F. H. L. Koppens, *Nano Lett.* **2019**, 19, 2765; b) G. Auton, D. B. But, J. Zhang, E. Hill, D. Coquillat, C. Consejo, P. Nouvel, W. Knap, L. Varani, F. Teppe, J. Torres, A. Song, *Nano Lett.* **2017**, 17, 7015.
